# Resistance to atrial fibrillation domestication and mitochondrial dysfunction in sheep: a potential key role of the TCA cycle and mitochondrial redox state

**DOI:** 10.1016/j.redox.2026.104391

**Published:** 2026-09-10

**Authors:** Guido Caluori, Stanley Nattel, Benoît Pinson, Patrice Naud, Bertrand Beauvoit, Stéphane Claverol, Fanny Vaillant, Sabine Charron, Florent Guilloteau, Virginie Loyer, Marion Constantin, Miruna Popa, Andrei Belykh, Anaïs Rogowsky, Andreas Haeberlin, Sylvain Ploux, Hassan Adam Mahamat, Rémi Dubois, Bastien Guillot, Philipp Krisai, Tsukasa Kamakura, Olivier Bernus, Pierre Jaïs, Pierre Dos Santos, Philippe Pasdois

**Affiliations:** a Univ. Bordeaux, INSERM, CRCTB, U 1045, IHU Liryc, Bordeaux, F-33000, France; b CHU de Bordeaux, INSERM, U 1045, Bordeaux, F-33000, France; c Department of Medicine and Research Center, Montreal Heart Institute and Université de Montréal, Montréal, Québec, Canada; d Service d’Analyses Métaboliques- TBMCore, CNRS UAR 3427, Inserm US 005, Univ. Bordeaux, France; e Univ. Bordeaux, INRAE, BFP, UMR 1332, Villenave d'Ornon, F-33140, France; f Plateforme Protéome, Univ. Bordeaux, Bordeaux, France; g Laboratoire de diagnostic moléculaire, hôpital Maisonneuve-Rosemont, Montréal, Québec, Canada; h Univ. Bordeaux, CNRS, IBGC, UMR 5095, Bordeaux, F-33000, France; i Department of Pharmacology and Therapeutics, McGill University, Montréal, Québec, Canada; j Institute of Pharmacology, West German Heart and Vascular Center, University of Duisburg-Essen, Essen, Germany

**Keywords:** Atrial fibrillation, Large animal models, Mitochondria, TCA cycle, Succinate

## Abstract

**Background:**

Atrial fibrillation (AF) often progresses from paroxysmal to more stable forms. It is well-recognized that patients vary in their AF progression, but the underlying mechanisms remain unclear. This work, performed in a sheep AF-model, aimed to identify atrial redox and energetic status differences between animals developing stable AF (AF-S) versus those resistant to AF-stabilization (AF-R).

**Methods:**

AF was induced with bursts of atrial tachystimulation, maintained whenever sinus rhythm resumed, and monitored with telemetry. Electrophysiological and structural remodeling were assessed *via* contact mapping, and histology, respectively. Proteomic, metabolomic, enzymatic, and bioenergetic remodeling were evaluated using left atrial appendage (LAA) tissues and isolated LAA mitochondria. Healthy rats were used to investigate whether a metabolic challenge stabilizes electrically induced AF episodes.

**Results:**

AF-S (N = 12) sheep developed stable AF after 13 days on average, whereas AF-R (N = 8) sheep failed to develop self-sustained AF despite 120 days of tachypacing. Contact mapping and histological analysis revealed that AF-S animals presented reduced atrial conduction velocity, and increased endomysial fibrosis. Metabolic analysis showed significant differences in tricarboxylic acid cycle (TCA) enzymes activities and a 45% succinate content increase in AF-S LAA versus AF-R. AF-S mitochondria showed abnormal succinate oxidation, decreased ATP synthesis rate, and increased ROS emission. The ratios of ATP/ADP, NAD^+^/NADH, and Complex I/II activities were disturbed in AF-S compared to AF-R. Calculated mitochondrial NAD^+^/NADH ratios suggest an oxidized state in AF-S while AF-R showed a reduced state. Exogenous succinate was metabolized when incubated with rat atrial cardiomyocytes, while intravenous succinate injection stabilized atrial arrhythmias electrically-induced *in vivo*.

**Conclusions:**

AF-S sheep showed TCA cycle, energetic and redox adaptations compared to AF-R. In this animal model, TCA cycle remodeling and associated redox and energetic responses determined the resistance to AF domestication, with potential relevance to identify new mechanistic determinants of AF progression in humans.

## 1 Introduction

Atrial fibrillation (AF) leads to significant population morbidity and mortality [1]. Allessie et al. provided the paradigm-shifting observation that AF episodes produce progressive atrial remodeling with the eventual development of spontaneously persisting AF in goats [2]. In patients, the transition rate from paroxysmal to permanent AF has been reported to vary between 15% to more than 50% within a follow-up period ranging from 1 to 12 years [[3], [4], [5], [6], [7]]. These studies show that many patients with paroxysmal AF never progress to persistent AF. The biological mechanisms behind this AF resistance remain poorly understood, despite significant clinical importance.

Studies on human atria have revealed metabolic alterations with AF, including changes in the mitochondrial electron transport chain [[8], [9], [10]], increased reactive oxygen species (ROS) production [11,12], altered glucose and fat metabolism [9,13], and oxidative damage to mitochondrial DNA [14]. Tricarboxylic acids (TCA) cycle intermediates, such as succinate, α-ketoglutarate, and malate have been correlated with subclinical atrial dysfunction, cardioembolic stroke, as well as AF [15,16]. Recently, chronic systemic succinate overload in mice was mechanistically linked to pro-arrhythmogenic atrial remodeling [17]. Whether individual differences in the metabolic response and endogenous accumulation of TCA cycle intermediates are linked to AF sensitivity or resistance is unknown.

To study the mechanisms of differences in resistance to AF development in an animal model, we implemented a sheep model of AF induced by intermittent rapid atrial pacing (RAP) [18,19], similar to Allessie's initial approach in goats [2]. We observed that a fraction of the instrumented sheep, despite 4 months of RAP exposure without loss of capture, never developed self-sustained AF ("AF resistant group", AF-R), while the majority did develop self-sustained AF ("AF sensitive group", AF-S), within three weeks of RAP onset. We tested the hypothesis that specific mitochondrial metabolic adaptations play a key role in determining resistance to AF.

## 2 Methods

### 2.1 Sheep AF model

This study used a modified version of the atrial fibrillation (AF) model described by Jalife and colleagues [18]. Sheep were prepared for the surgical procedure after an intramuscular injection of ketamine (20 mg/kg), acepromazine (0.1 mg/kg) and buprenorphine (9 μg/kg). Anesthesia was induced by an intravenous bolus dose of propofol (1 mg/kg) *via* a venous catheter in the foreleg, followed by endotracheal intubation and ventilation with a 1:1 mixture of room air and oxygen containing isoflurane (1.5 to 3%, as needed to maintain deep anesthesia). Thirty-one female sheep (Charmoise, mean age = 8.4 ± 0.1 years, mean weight = 50.8 ± 1.2 kg) were implanted with a dual chamber pacemaker (St. Jude Medical, Inc, St Paul, MN), two pacing leads (IS1 leads Tendril STS 2088 TC, St. Jude Medical, Inc, St Paul, MN), and a telemetry device (EasyTEL + L – ETA, Emka Technologies, Paris, FR). After 7 days of recovery, the pacemaker was randomly activated to either sense and record the right atrial and ventricular signals (Sham group, N = 11) or to sense, record and pace the right atria (Supplemental Fig. 1A–C) to induce AF (AF-induction group, N = 20). In the AF-induction group, bipolar square-wave atrial stimuli at 5-times voltage threshold and 10-Hz frequency were used to induce AF whenever sinus rhythm (SR) was detected. Tachypacing was delivered for 30 s, after which a 7-s pause was allowed to determine the spontaneous rhythm. Telemetry data were analyzed in Matlab 2022.a (Mathworks) for AF burden and in LabChart v8 (ADInstruments) for pseudo-ECG analysis (see online supplemental files for details on model development and access to raw telemetric data).

### 2.2 Atrial endocardial electrical contact mapping

Electroanatomical mapping was performed with a CARTO™ 3 system using CARTO™ v6 software (Biosense Webster, Irvine, CA, USA), and a 20-pole steerable mapping catheter (PENTARAY™ NAV, Biosense Webster). Conduction velocity, (CV) and local effective refractory period (ERP) were calculated in the right and left atrium with programmed electrical pacing. Sinus rhythm was restored by direct-current cardioversion (1 shock, 50-70J) of AF-S animals prior to contact mapping.

### 2.3 Sheep euthanasia and heart explantation

After anesthesia (see above) a median thoracotomy was performed and the heart was exposed. Mechanical activity was stopped by aortic retroperfusion with ice-cold Ringer-Lactate solution at a pressure of 80 mmHg, and by filling the chest cavity with ice-cold NaCl solution (9 g/L). After complete loss of mechanical activity, disappearance of a detectable arterial pressure and total absence of response to pain, the heart was explanted.

### 2.4 Sheep atrial histology and structural characterization

The left atrial posterior wall was fixed in 4% paraformaldehyde in phosphate-buffered saline. Thin paraffin-embedded sections (5-μm) were stained with Masson trichrome. The slides were scanned with an Axio Scan.Z1 microscope (ZEISS, Jena, Germany) with 20x magnification in brightfield and the tiles automatically assembled to form the section overview. The images were imported into Fiji [20] for calculation of endomysial fibrosis, cardiomyocyte cross-section area and cell-to-cell distance.

### 2.5 Proteomics

*nLC-MS/MS analysis and Label-Free Quantitative Data Analysis*. Following sample preparation and protein digestion (see Methods in Online Supplement), peptide mixture was analyzed on an Ultimate 3000 nanoLC system (Dionex, Amsterdam, Netherlands) coupled to an Electrospray Orbitrap Fusion™ Lumos™ Tribrid™ Mass Spectrometer (Thermo Fisher Scientific, San Jose, CA). Separation flow-rate was set at 300 nL/min. The mass spectrometer operated in positive-ion mode at a 2-kV needle voltage. Data were acquired using Xcalibur 4.4 software.

*Proteomic analysis.* Data were searched by SEQUEST through Proteome Discoverer 2.5 (Thermo Fisher Scientific Inc.) against an *Ovis aries* uniprot database (21,195 entries in v2022-04). Spectra from peptides higher than 5000 Da or lower than 350 Da were rejected. Precursor Detector node was included. Peptide validation was performed using Percolator algorithm [21] and only “high confidence” peptides were retained corresponding to a 1% False Positive Rate at peptide level. Peaks were detected and integrated using the Minora algorithm embedded in Proteome Discoverer. Normalization was performed based on total *Ovis aries* protein amount. Quantitative data were considered for proteins quantified by a minimum of two peptides and a statistical *p*-value lower than 0.05 after correction for multiple comparisons with the Benjamini-Hochberg method.

*Bioinformatics:* All statistical analyses of proteomic data were conducted using RStudio (Version 2026,06,1, RStudio, PBC), the online platforms Reactome [22] and Metaboanalyst 6.0 [23]. We maintained an R Project to organize scripts, datasets, and outputs systematically, with R version 4.2.2. Batch effects were corrected using the 'limma' package in R. Data manipulation was performed with the 'dplyr' package to streamline the datasets into structured formats amenable to downstream analysis. Proteins were annotated with biological pathways using Wikipathway database. The original data set and analysis is available on the following link: https://doi.org/10.6084/m9.figshare.28269872.v1.

### 2.6 Rat euthanasia and heart explant

Anesthesia was initially induced in male Wistar rats (225-300 g) with 3.5% isoflurane for 3 min. For heart explantation, rats were anticoagulated (heparin 1000 IU/Kg) and left in the induction chamber with 3.5% isoflurane for a further 3 min before euthanasia. After confirmation of the lack of pain reflex by pinching the lower paws and the tail, euthanasia was performed by cervical dislocation. The heart was removed after death confirmation by the absence of respiration.

### 2.7 Rat atrial cardiomyocyte (RACM) isolation

RACM were enzymatically dissociated by collagenase digestion from the atria of male Wistar rats (225-300 g, N = 10) after cardiac explant (see Online Supplement).

### 2.8 Steady-state ROS production in RACM

Freshly-isolated RACMs were allowed to sediment in Tyrode solution on a 35 mm Fluorodish (WPI, Sarasota, FL-US) at RT for 1h. They were then incubated for 30 min under 95% room-air/5% CO_2_ at 37°C in the presence of 5 μmol/L CellROX ™ Green (Thermo Fisher Scientific, MA-US). After incubation, brightfield and epifluorescence images were obtained with a Leica Thunder Imager system (Leica Camera, Wetzlar, Germany) and 488/530 excitation/emission filters. To observe the effect of exogenous succinate application on intracellular ROS production, RACMs were incubated with 5 mmol/L succinate (buffered with HEPES 25 mmol/L at pH 7.4 RT). RACMs were also incubated with Tyrode's solution and H_2_O_2_ (50 μmol/L), as negative and positive control respectively.

### 2.9 RACM incubation with exogenous succinate for metabolomic analysis

Freshly isolated RACM preparations (N = 4) were equally divided into two 1.5 ml conical vials each containing 1 ml of cell suspension. One vial was supplemented with succinate (buffered with HEPES 25 mmol/L at pH 7.4 RT) to reach a final concentration of 5 mmol/L. The paired vial was supplemented with Tyrode buffered with HEPES 25 mmol/L at pH 7.4 RT. Both vials were placed in an incubator at 37°C under gentle agitation for 30 min. Subsequently, a cell pellet was obtained by spinning the cell solution at 100 g for 3 min at 4°C. The supernatant was removed and the pellet was gently resuspended in fresh ice-cold Tyrode to dilute the residual succinate. This operation was repeated twice. The final dry pellet was snap frozen in liquid nitrogen and stored at −80°C for mass spectrometry analysis.

### 2.10 Rat Langendorff perfusion and surface fluorescence measurements

Hearts from male Wistar Rats (225-300 g, N = 5) were cannulated and perfused in Langendorff mode and an optic-fiber cable, connected to a modified spectrofluorometer (Xenius, SAFAS Monaco), was placed in front of the LAA [24]. The NAD(P)H pool redox state was assessed on the beating LAA by recording autofluorescence (340/460 nm). To observe mitochondrial H_2_O_2_ emission, hearts were loaded with Mitochondria Peroxy-Yellow 1 (3 μmol/L MitoPY1, 485/535 nm, Tocris Bioscience, #4428) [25]. Succinate (pH 7.4) was then perfused at 5 mmol/L with a syringe pump for 10 min. A second washout was then performed before starting H_2_O_2_ perfusion (400 μmol/L) for 15 min. Distance and motion artefacts were diminished by dividing the emitted photon intensity by reflected signals measured at the respective excitation and emission wavelengths [26].

### 2.11 In-vivo assessment of susceptibility to atrial arrhythmia in the rat

Anesthesia was induced in male Wistar rats (250-300 g, N = 20) with isoflurane (3.5%) and they were transferred supine to a heated mat equipped with an anesthesia mask (providing 80% oxygen/20% room-air with 2.5% isoflurane (Minerve, Esternay, France)). A CIB'ER Mouse® catheter (NuMED, Inc., Hopkinton, NY-USA) was introduced into the esophagus until an atrial electrogram was recorded. A 26G cannula inserted *via* the caudal vein was connected to a syringe pump for randomly assigned IV infusion of vehicle or succinate solution (80 mg/mL) at 15 mL/kg/h. In some experiments, 10 μmol/kg S1QEL1.1 was injected intraperitoneally at least 5 min prior to IV infusion. Atrial tachyarrhythmia (AF/AT) vulnerability was quantified 10 min after IV injection with 10 RAP bursts at 30 Hz for 30 s, spaced 5-10 s to visually assess the presence of SR or AF/AT. In a parallel set of experiments (N = 6 Wistar rats), the same *in vivo* atrial tachypacing protocol was applied and serum collected in order to perform a targeted metabolomic analysis by mass spectrometry. Rat serum was obtained after cannulation of the ventral tail artery, from which 1-2 ml of blood were drawn in a non-treated conical vial. The blood was allowed to clot at RT for 20 min, before being centrifuged twice at 2000 g, 4°C, 10 min. Serum aliquots were snap frozen in liquid nitrogen and stored at −80°C. For baseline paired serum collection 500 μL of blood was taken before starting the atrial tachypacing sequence.

### 2.12 Sheep LAA mitochondrial isolation and purification

Sheep LAA mitochondrial fraction was isolated and purified as previously published [27] with modifications. Following heart excision, the entire sheep LAA was dissected free and chopped into fine pieces before incubation at 4°C for 45 min while stirring in 30 mL of buffer containing (mmol/L): CaK_2_EGTA 2.77; K_2_EGTA 7.23 (pCa = 7); MgCl_2_ 6.56; dithiothreitol (DTT) 0.5; MES 50; imidazole 20; taurine 20; Na_2_ATP 5.3; creatine phosphate 15, and 1 mg/mL collagenase type-2 (Worthington, Lakewood, NJ-US); pH 7.3 at 4°C with KOH. The resulting tissue suspension was used to isolate and purify mitochondria as previously described [27]. The mitochondrial suspension, composed of interfibrillar and subsarcolemmal mitochondria, was kept on ice at 80-100 mg/mL (Biuret assay) for < 6 h pre-study or frozen in liquid-N_2_ and stored at −80°C for later biochemical analysis.

### 2.13 *Measurements of respiration, H*_*2*_*O*_*2*_*emission, NAD(P)H autofluorescence, and ATP synthesis in isolated sheep LAA mitochondria*

Mitochondrial function was studied in different bioenergetic states: basal (respiratory substrates only, state 2), state 3 (ADP 1.5 mmol/L) and after ATP-synthesis inhibition (state 4, carboxyatractlyloside, CAT, 5 μmol/L). Oxygen-consumption was measured by oxygraphy at 37°C (Digital Model 10, Rank Brothers and PowerLab, ADInstruments, Sidney, Australia). Mitochondria (0.25 mg/mL) were added to a buffer containing (mmol/L): mannitol 40, KCl 100, EGTA 1, MgCl_2_ 20, KH_2_PO_4_ 10, Amplex® Red 15 μmol/L, horseradish peroxidase 12 μg/ml, and respiratory substrates. The respiratory chain was fueled with different substrate combinations, (i) GMS: glutamate (5 mmol/L) + malate (2 mmol/L) + succinate (5 mmol/L), (ii) GMS + S1QEL1.1 (210 nmol/L) (iii) PCM: palmitoyl-carnitine (15 μmol/L) + malate (2 mmol/L), or (iv) PM: pyruvate (5 mmol/L) + malate (2 mmol/L). Oxygen-consumption rates are expressed in natO/min/mg protein. The cap of the oxygraphic cell was equipped with an optic fiber coupled to a fluorometer (Xenius, Safas Monaco), and with a glass pH microelectrode (ThermoFisher Scientific). Autofluorescence of NAD(P)H (340/460 nm) and resorufin (570/600 nm) fluorescence were collected through the optic fiber. During ATP-synthesis, pH increase was converted into H^+^-consumption after HCl titration (Titripur Reagents, Merck). H^+^-consumption flux was expressed in nmol H^+^/min/mg protein and converted into ATP-synthesis flux, expressed in nmol of ATP/min/mg protein according to published methodology [28,29]. Oxidative phosphorylation efficiency, P to O ratio, was expressed as the ratio of maximal ATP-synthesis to oxygen consumption rates measured during ATP-synthesis. The inner membrane coupling was expressed as the ratio of state 3 to state 2 respiration.

### 2.14 Measurement of sheep LAA mitochondrial membrane potential

Mitochondrial membrane potential (ΔΨ_m_) was measured with a tetraphenylphosphonium (TPP^+^)-sensitive electrode (O2k, OROBOROS Instruments, Austria). Calibration was performed before each run with known quantity of TPP^+^ (2 μmol/L maximum according to published methodology [30]) in the respiration buffer supplemented with respiratory substrates (PM, GMS or PCM). ΔΨ_m_ was quantified according to published methodology [31] with respiratory substrates only, after ADP addition (1.5 mmol/L) followed by CAT (5 μmol/L) on a solution containing 0.2 mg/mL mitochondria. The mitochondrial matrix volume was set to 1.1 μL/mg [32]. Non-specific TPP^+^ mitochondrial binding was corrected for by adding 2 μmol/L carbonyl cyanide 4-(trifluoromethoxy)phenylhydrazone (FCCP) at the end of each run.

### 2.15 Sample preparation for biochemical analysis

*Metabolite extraction:* Metabolites were either extracted with an acidic methodology or using an ethanol boiling methodology adapted from Ref. [33]. For the acidic approach, pieces of *in vivo* freeze-clamped LAA were pulverized under liquid-N_2_, and 100-150 mg were mixed with 500 μL cold perchloric acid (10%) solution supplemented with EDTA (10 mmol/L) and neutralized (pH 7.2-7.4 with KHCO_3_ and KOH-MOPS). For the ethanol-based methodology each rat plasma sample (100 μL) was mixed with 900 μL of extraction buffer (ethanol/HEPES 0.1 mM, pH 7.0, in a 4:1 ratio). For rat atrial cardiomyocyte pellets and sheep LAA frozen powder, samples were resuspended in 1 mL of the same extraction buffer and sheep LAA were homogenized using a 5 ml glass/PTFE Potter-Elvehjem grinder. For rat plasma and atrial cardiomyocytes, succinic acid-2,2,3,3-D_4_ (a deuterium-labelled succinic acid in which the four methylene hydrogens are replaced by deuterium; #293075, Sigma-Aldrich) was added to the biological extracts at a final concentration of 1 nmol/mL in the extraction buffer. This compound was utilized as an internal standard for normalization during mass spectrometry analyses. All biological materials were subsequently heated at 80°C for 3 min and dried using a rotavapor (140 rpm) at 65°C for 3 min. The resulting residues were reconstituted in either 1 mL of pure water (for plasma), 400 μL (for atrial cardiomyocytes), or 10 μL/mg tissue (for sheep LAA). Insoluble material was removed by centrifugation at 21,000g for 1 h at 4°C. The clarified supernatant was subjected to ultrafiltration using Nanosep 10K Omega filters (#OD010C35, Pall).

To evaluate specific activities of cytosolic enzymes, 50-100 mg of frozen pulverized LAA were mixed with 200-400 μL of buffer containing 50 mmol/L KH_2_PO_4_, 50 μmol/L DTT, 0.3% w/v Tx-100, protease inhibitors (Roche cOmplete™), pH 7.2 at RT. For mitochondrial enzymes activity assessment, frozen isolated LAA mitochondria were solubilized in the same buffer at a concentration ranging from 0.2 to 4 mg/ml depending on the enzymes studied.

### 2.16 Sheep LAA metabolite content assessment

LAA metabolites content analysis was performed either by Liquid Chromatography Coupled to Conductimetry, UV absorbance or Mass Spectrometry Detectors or by enzymatic quantification from *in vivo* freeze-clamped LAA tissue. The detailed methodology is available in the online supplement.

### 2.17 Assessment of mitochondrial and cytosolic enzyme specific activities

All specific activities were measured at 37°C by spectrophotometry (V-760, JASCO UK Limited) or fluorimetry (FP-8500, JASCO UK), unless otherwise stated. The detailed procedure for enzymatic assay is available in the online supplement. The following enzymes activities were assessed on isolated LAA mitochondria: NADH:decylubiquinone oxidoreductase (Complex I), succinate:ubiquinone oxidoreductase (Complex II), pyruvate dehydrogenase (PDH), alpha-ketoglutarate dehydrogenase (*α*-KGDH), succinyl-CoA synthetase (SCS), fumarase, malate dehydrogenase isoform 2 (MDH2), and D/L hydroxyglutarate dehydrogenase (2-HGDH). Citrate synthase (CS) specific activity was assessed on atrial homogenates to evaluate mitochondrial content variation between groups.

### 2.18 Measurement of carbonyl content in sheep LAA tissue and rat heart

*Spectrophotometric methodology in sheep atrial and rat heart samples:* 60-80 mg of sheep LAA or rat heart frozen tissue powder were solubilized in guanidine solution (6 mol/L). 200 μL of homogenate were mixed with 800 μL of 12.5 mmol/L 2,4-Dinitrophenylhydrazine (DNPH) solution or with 800 μL of 2.5 mol/L HCl and shaken for 1 h in the dark. Following incubation, proteins were precipitated with 2 cycles of trichloroacetic acid treatment at 20 and 10%. Protein pellets (10,000 g 10 min) were washed three times, with 50/50 ethyl-acetate-ethanol solution. The final pellets were resuspended in 300 μL guanidine solution (6 mol/L). Sample absorbance was assessed at 370 nm (Xenius, SAFAS Monaco). Protein concentration was determined using a bicinchoninic acid assay (ThermoFisher Scientific, #23227). Total protein carbonyl content was calculated considering the background absorbance and expressed in nmol/mg protein.

*Western-Blot methodology on rat heart samples:* Aliquots of 30 μg total protein previously extracted from the frozen rat heart tissue powder were diluted in a 15 μL volume of 6% (wt/v) SDS. An equal amount of 15 μL of 20 mM DNPH in 10% (v/v) trifluoroacetic acid was added into the protein solution and incubated at RT for 10 min. The solution was then neutralized with 15 μL of 2 M Tris in 30% (v/v) glycerol, containing 7% (v/v) β-mercaptoethanol. The reacted protein solution was then processed for conventional Western Blot analysis, using goat anti-2,4-dinitrophenyl (DNP) as primary antibody (1:5000, A150117A, Bethyl Laboratories, Inc.), and Rabbit Anti-Goat IgG (H + L)-HRP Conjugate (1:5000 dilution, 1721034, Bio Rad Laboratories).

### 2.19 qPCR in sheep LAA tissue

RNA was extracted from frozen LAA tissue using the RNAeasy Universal Kit (Qiagen) as per manufacturer's instructions. Extracted RNA was mixed with RNase-free water and Owi Script Reverse Transcriptase UltraMix (5X) to a final concentration of 12 ng/μg and then put in the thermocycler for reverse transcription. For the qPCR reaction mix, primers initially stored at 10 μmol/L were diluted to the optimized working concentration of 300, 600 or 800 nmol/L (Online Supplemental Table 3) and mixed with SYBR Green Supermix (2X) and RNA-free water. Each triplicate sample was placed into a 96-well plate with a volume of 1 μL and mixed with 9 μL of reaction mix. After they were spun down, plates were placed in the thermocycler for qPCR in sheep LAA tissue. Raw data were normalized to housekeeping genes (b2m and HPRT1) expression with Bio-Rad CFX Manager software. These values were then expressed relatively to the Sham group using the geometric mean for normalization.

### 2.20 Metabolite compartmentation analysis at equilibrium

The [NAD^+^] to [NADH] ratio, oxaloacetate and alpha-ketoglutarate concentration within the cytosolic and mitochondrial compartments were calculated assuming that the system was at thermodynamic equilibrium. The same approach was used to calculate the proton motive force (Δp) and tissue [NAD^+^] to [NADH] ratio. The equations and values used for the ΔG°’ calculation are presented in the online supplement.

### 2.21 Statistical analysis

All statistics were performed using GraphPad Prism. Data are presented as mean ± SD. Comparison were performed with non-parametric tests, because (i) the number of individual experiments varied from 3 to 13, (ii) these tests can be used in presence of outliers (the sheep model displaying important variability for some parameters, especially in the Sham group), and (iii) evaluation of normality and homoscedasticity of individual samples are not required. For comparison of multiple independent samples or two independent samples, the non-parametric Kruskal-Wallis test with Dunn's correction for multiple comparisons and non-parametric multiple t-tests with the Holm-Sidak correction for multiple comparisons were used, respectively. For comparison of two groups of unpaired or paired samples, Mann-Whitney or Wilcoxon tests were used respectively. For survival curve analysis, we used the Kaplan-Meyer model. Differences were considered significant for a *p*-value <0.05; exact *p* values are shown in figures when significant and if not “ns” was added on the figure. The number of independent biological experiments is indicated directly on the figure by the number in brackets.

## 3 Results

### 3.1 LAA electrical and structural remodeling

Among the animals in the AF-induction branch, two groups of sheep emerged, one developing stable self-sustained AF (AF-S, N = 12), and one resistant to AF progression and stabilization (AF-R, N = 8).

**Fig. 1:**
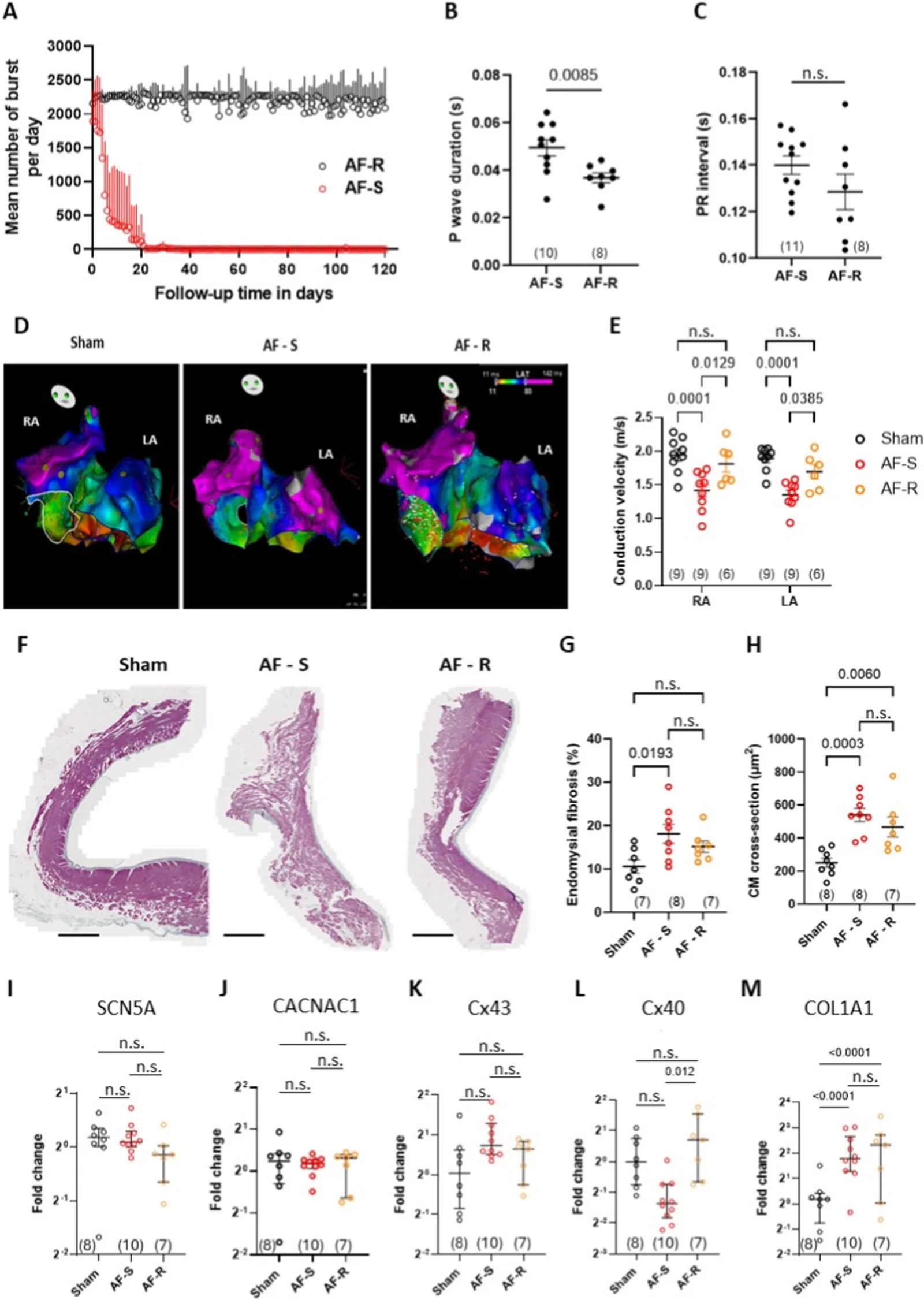
**AF properties, electrophysiological and structural remodeling**. **A**: Tachypacing bursts per 24-h as a function of follow-up time. Results shown for AF-Sensitive (AF-S, N = 12) and AF-Resistant (AF-R, N = 8) sheep. **B-C**: P wave duration (B) and PR interval (C) recorded before starting the RAP protocol. **D**: Pseudo left anterior oblique (LAO) view of representative electro-anatomical maps of the bi-atrial local activation time, for the three groups (RA, right atrium, LA, left atrium). Scalebar range is 11-80 ms for all maps and groups. **E**: Atrial conduction velocity calculated from the 3D maps obtained during contact mapping of the right (RA) and left (LA) atrium. **F**: Representative images of left atria (LA) posterior wall histological preparations, for the three groups, after Masson trichrome stain and scanning. Scale bar = 2 mm. **G**: Endomysial fibrosis measured from histological sections stained with Masson Trichrome. **H**: CM cross-sectional area. **I**-**M**: LAA tissue fold change of mRNA transcripts expression level of Sodium channel protein type 5 subunit alpha (Scn5a, **I**), Voltage-dependent L-type calcium channel subunit alpha-1C (Cacna1c, **J**), Connexin 43 (Cx43, **K**), Connexin 40 (Cx40, **L**), and Collagen type I alpha-1 chain (COL1A1, **M**). Panels B, C, E, G, H and I to M show individual points and mean ± SD. Statistical analysis was performed with Kruskal-Wallis's test and Dunn's post-hoc test for multiple comparisons. Complementary analysis is presented in Supplemental Fig. 1.

AF-S sheep developed self-sustained AF (defined by episodes >24 h) after an average of 13 days (range 5-22, Fig. 1A and Supplemental Fig. 1A), while AF-R sheep never did along the follow-up window (Fig. 1A and Supplemental Fig. 1B). No adverse events were observed that required changes to the pacing protocol (Supplemental Fig. 1C). Telemetry showed that 60% of AF-S sheep maintained sustained AF with no bursts required during the follow-up period, while the remaining 40% received between 1 and 21 bursts (Supplemental Fig. 1D) during the entire follow-up period, representing less than 0.1% of daily time in sinus rhythm during the stable AF period. In contrast, AF-R sheep received on average 2300 bursts per 24 h and never showed self-sustained AF (Supplemental Fig. 1B). Pseudo-ECG analysis before the start of the tachypacing protocol showed that AF-S animals presented a significantly longer P-wave duration, but no difference in PR interval (Fig. 1B and C respectively). At the end of the follow-up period, AF-S animals showed a significantly longer P wave duration (Supplemental Fig. 1F), compared with the other groups, whilst both treatment groups presented a trending increase of PR interval when compared to Sham (Supplemental Fig. 1G).

Contact mapping at the final electrophysiological study (Fig. 1D) and programmed stimulation, performed under isoflurane anesthesia, showed similar ERP reduction in both groups compared to Sham animals (Supplemental Fig. 1E), but distinctive reduction in right/left atrium conduction velocity (CV) for AF-S animals (Fig. 1E). Endomysial fibrosis was significantly increased in AF-S versus Sham only (Fig. 1F and G), while AF-S and AF-R sheep both had increased cardiomyocyte cross-sectional area versus Sham (Fig. 1H), with no change in intercellular spacing (Supplemental Fig. 1H). A targeted mRNA analysis *via* qPCR showed no significant changes in mRNA expression for subunits of sodium (Fig. 1I, SCN5A), calcium (Fig. 1J, CACNA1C), potassium (Supplemental Fig. 1I, KCNJ11 and 1J, KCNN2) ion channels, or Cx43 (Fig. 1K). Importantly, we did observe a distinct reduction of Cx40 transcription for AF-S vs AF-R animals (Fig. 1L), and COL1A1 transcription was increased for both groups vs Sham (Fig. 1M). Nonetheless, Cx40 quantification by Western-Blot showed an increased expression in AF-R compared to AF-S (Supplementary Fig. 1K and 1L).

Thus, both AF-S and AF-R shared common features of electrophysiological and structural remodeling, with AF-S sheep being characterized by a significantly slower atrial CV that likely contribute to AF stabilization. To further characterize the differences between AF-S and AF-R, we then assessed the extent of metabolic remodeling.

### 3.2 Proteomic, enzymatic and metabolomic profiling

**Fig. 2:**
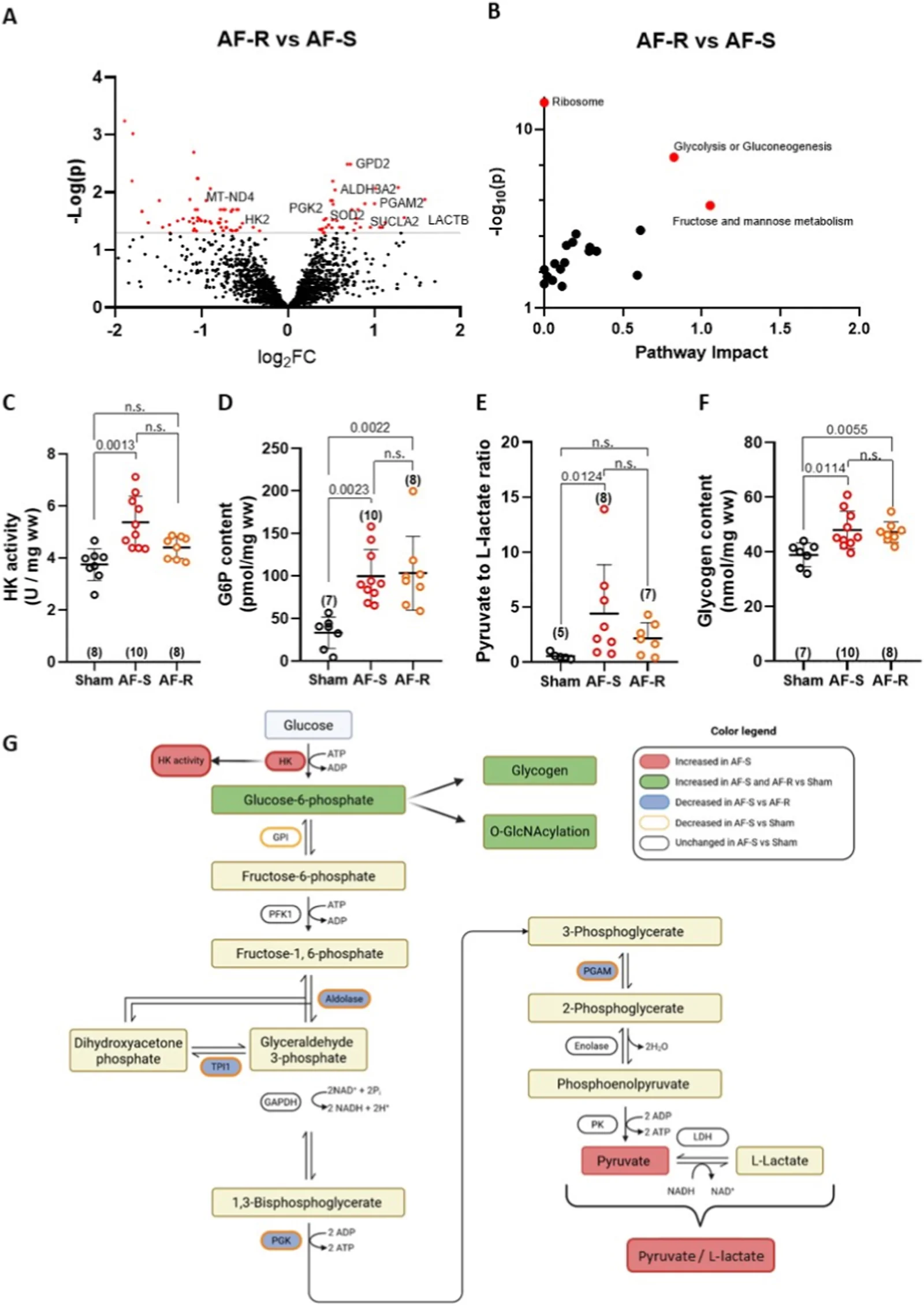
**Proteomic, enzymatic and metabolomic analysis of LA-appendage**. LAA samples freeze-clamped *in vivo* were analyzed by mass spectrometry for proteins identification. The computational analysis suggested dysregulation of multiple proteins involved in mitochondrial function, metabolism and bioenergetics. **A**: Volcano-plot obtained by comparing AF-R to AF-S sheep. **B**: Pathway enrichment analysis results for the comparisons between AF-R and AF-S – red dots represent the pathways with false discovery rate <0.05. **C** to **F**: Hexokinase activity (HK, panel **C**), Glucose-6-phosphate (G6P, panel **D**), pyruvate to l-Lactate ratio (panel **E**), and Glycogen content (panel **F**) assessed in frozen LAA. Panel **G**: scheme summarizing the glycolytic remodeling assessed by proteomic, enzymatic and metabolomic studies. Complementary results are shown in supplemental figures 2, 3 and 4. **C** to **F** show individual points and mean ± SD. Statistical analysis was performed with Kruskal-Wallis test and Dunn's post-hoc test for multiple comparisons. Numbers in brackets correspond to individual experiments for each group.

LAA proteomic profiling revealed significant differences between Sham, AF-S and AF-R sheep (Fig. 2A and B, Supplemental Fig. 2, Supplemental Table 4, data set available at: https://doi.org/10.6084/m9.figshare.28269872.v1). Both AF-S and AF-R exhibited increased expression of proteins involved in ribosome assembly versus Sham (Supplemental Fig. 2A and 2B), suggesting a common pattern of altered proteostasis. However, they also presented distinctive features: AF-R showed significant alterations in pathways involved in adrenergic signaling and cardiac contraction, while AF-S primarily exhibited modulation of metabolic pathways, including glycolysis/gluconeogenesis, fatty acid degradation, pyruvate and branched chain amino acid metabolism (Fig. 2A–B, Supplemental Table 4). Compared to AF-R, AF-S sheep showed consistent remodeling of the glycolytic pathway, with a notable overexpression of hexokinase-2 (HK2, Supplemental Table 4) and downregulation of TCA cycle enzymes namely fumarase (FH) and the ADP sensitive subunit of succinyl-CoA synthase (SUCLA2, Supplemental Table 4).

Based on these results we quantified LAA tissue specific activity of hexokinase (Fig. 2C) and l-lactate dehydrogenase (LDH, Supplemental Fig. 2C). We also quantified G6P (Fig. 2D), pyruvate (Supplemental 2D), l-lactate (Supplemental Fig. 2E), pyruvate to l-lactate ratio (Fig. 2E), and glycogen (Fig. 2F) content in LAA. These results confirm that the glycolysis pathway is modified in both AF-S and AF-R groups versus Sham, promoting G6P and associated glycogen accumulation in both groups. A schematic summarizing the glycolytic remodeling in the three groups is shown in Fig. 2G. G6P is also linked to posttranslational modification (PTM) by O-GlcNAcylation, a PTM proposed to participate in AF-progression in the context of diabetes [34]. Assessment of protein O-GlcNAcylation in AF-S and AF-R (Supplemental Fig. 3A) showed a net increase when compared to Sham, with no changes in the expression of the three enzymes regulating this pathway (Supplemental Figs. 3B and 4). Modulations in the glycolytic pathway in AF-S and AF-R groups were not associated with increased activity or expression level of LDH (Supplemental Fig. 2C and https://doi.org/10.6084/m9.figshare.28269872.v1) or accumulation of l-lactate (Supplemental Fig. 2E) compared to Sham. Only the AF-S group showed both an accumulation of pyruvate (Supplemental Fig. 2D) and an increased pyruvate to l-lactate ratio (Fig. 2E) versus Sham. Finally, the AF-R group showed an increased expression of the monocarboxylate isoform 1 (MCT1) when compared to Sham (Supplemental Fig. 2F).

Compared to AF-R, the proteomic profile of AF-S LAA tissue (Supplemental Table 4), was characterized by a significant decrease in TCA cycle enzymes (FH, SUCLA2), cytosolic and mitochondrial adenylate kinases (AK1, AK2), ROS detoxification (SOD2, PRDX3), cellular and mitochondrial detoxification (ALDH3A2, ALDH2), beta-oxidation enzymes (HADH), and mitochondrial glycerol-3-phosphate metabolism (G3P2). AF-S LAA also showed increases in protein expression related to hypertrophic signaling (STAT3), mitochondrial aldehyde detoxification (ALDH2), glutathione synthesis (GCLC), mitochondrial outer membrane fusion (MFN1), complex I subunit (MT-ND4) (Supplemental Table 4, raw data available at https://doi.org/10.6084/m9.figshare.28269872.v1).

**Fig. 3:**
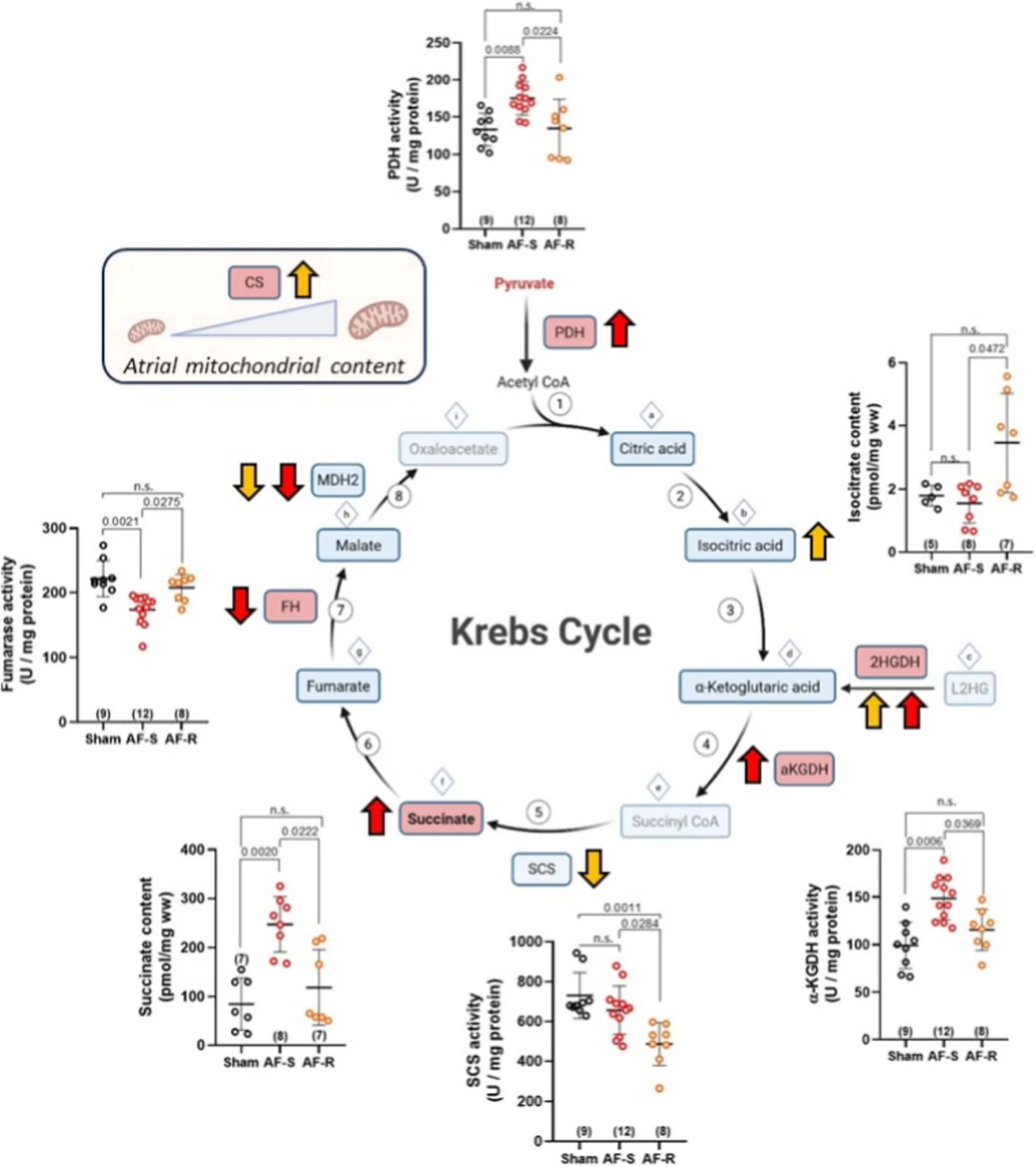
**Metabolite content and enzyme activity in LA Appendage (LAA).** Samples of LAA freeze-clamped *in vivo* were analyzed by high-pressure ion chromatography (HPIC) or enzymatic assay for metabolites belonging to the TCA cycle (see methods). Metabolite content is expressed in pmol/mg wet weight (ww). Enzymes' activity was assessed on isolated mitochondria except for Citrate Synthase (CS) which was analyzed in LAA homogenate to evaluate mitochondrial content. In accordance to the graph plots red and orange arrows represent the changes in metabolites or enzyme activity in the AF-S and AF-R, respectively. For TCA cycle intermediates, citrate (a), isocitrate (b), α-Ketoglutarate (c), succinate (f), fumarate (g), and malae (h) content were assessed. For mitochondrial enzymes, Pyruvate dehydrogenase (PDH), alpha-ketoglutarate dehydrogenase (*α*-KGDH, 4), fumarase (FH, 7), succinyl-CoA synthetase (SCS, 5), l-malate dehydrogenase isoform 2 (MDH2, 8), and D/L-2-hydroxyglutarate dehydrogenase (2HGDH). Acetyl-CoA, Oxaloacetate and succinyl-CoA could not be detected in our samples. Red arrows and orange arrows show the changes in AF-S and AF-R, respectively. Panels show individual data points and mean ± SD. Statistical analysis was performed with Kruskal-Wallis test and Dunn's post-hoc test for multiple comparisons. Numbers in brackets correspond to individual experiments. Complementary figures are presented in Supplemental Fig. 5.

Given these findings, targeted metabolomic analysis for TCA cycle metabolites (atrial tissue homogenate) and screening of the activity of TCA cycle enzymes (isolated LAA mitochondria) were performed to further explore mitochondrial metabolic differences between the three groups. The results are summarized in Fig. 3 and Supplemental Fig. 5. Compared to AF-S, AF-R showed a significant increase in isocitrate content, while citrate and l-malate content showed no differences (Supplemental Fig. 5A and 5B). Compared to AF-R, the AF-S group showed an accumulation of succinate (Fig. 3), a substrate of respiratory chain complex II, while no differences in fumarate were observed between groups. Regarding TCA enzyme activity assessment, citrate synthase activity was evaluated at the tissue level and revealed increased activity in AF-R versus Sham, while AF-S sheep showed an intermediate phenotype (N.S. vs AF-R or Sham, Supplemental Fig. 5C). At the level of isolated mitochondria, the AF-S group was characterized by increased activity of pyruvate dehydrogenase (PDH, Fig. 3) and alpha-ketoglutarate dehydrogenase (*α*-KGDH, Fig. 3), while fumarase activity was decreased (Fig. 3). Both AF-S and AF-R mitochondria showed decreased activity of l-malate dehydrogenase (MDH2, Fig. 3 and Supplemental Fig. 5D), and increased activity of 2-hydroxyglutarate dehydrogenase (Fig. 3 and Supplemental Fig. 5E), an enzyme that produces alpha-ketoglutarate. In contrast, AF-R showed a decrease in succinyl-CoA synthase activity (SCS, Fig. 3) compared to both Sham and AF-S, in agreement with the decreased expression of the SUCLA2 subunit of this enzyme that we noted (Supplemental Table 4 and https://doi.org/10.6084/m9.figshare.28269872.v1).

These integrated proteomic, metabolomic, and enzymatic findings suggest that mitochondrial and metabolic remodeling are associated with AF resistance and provided the impetus to further investigate mitochondrial function in isolated mitochondria from LAA tissue of AF-S and AF-R sheep.

### 3.3 LAA mitochondrial function assessment

We designed experiments to assess mitochondrial function using various respiratory substrates (state 2: non-phosphorylating respiration) and after maximal ATP synthesis induction (state 3: phosphorylating respiration), while mitochondria were oxidizing pyruvate + malate (PM), or glutamate + malate + succinate (GMS), or GMS along with the succinate-driven ROS emission inhibitor S1QEL1.1 [35] (S1QEL), or palmitoyl-carnitine + malate (PCM).

**Fig. 4:**
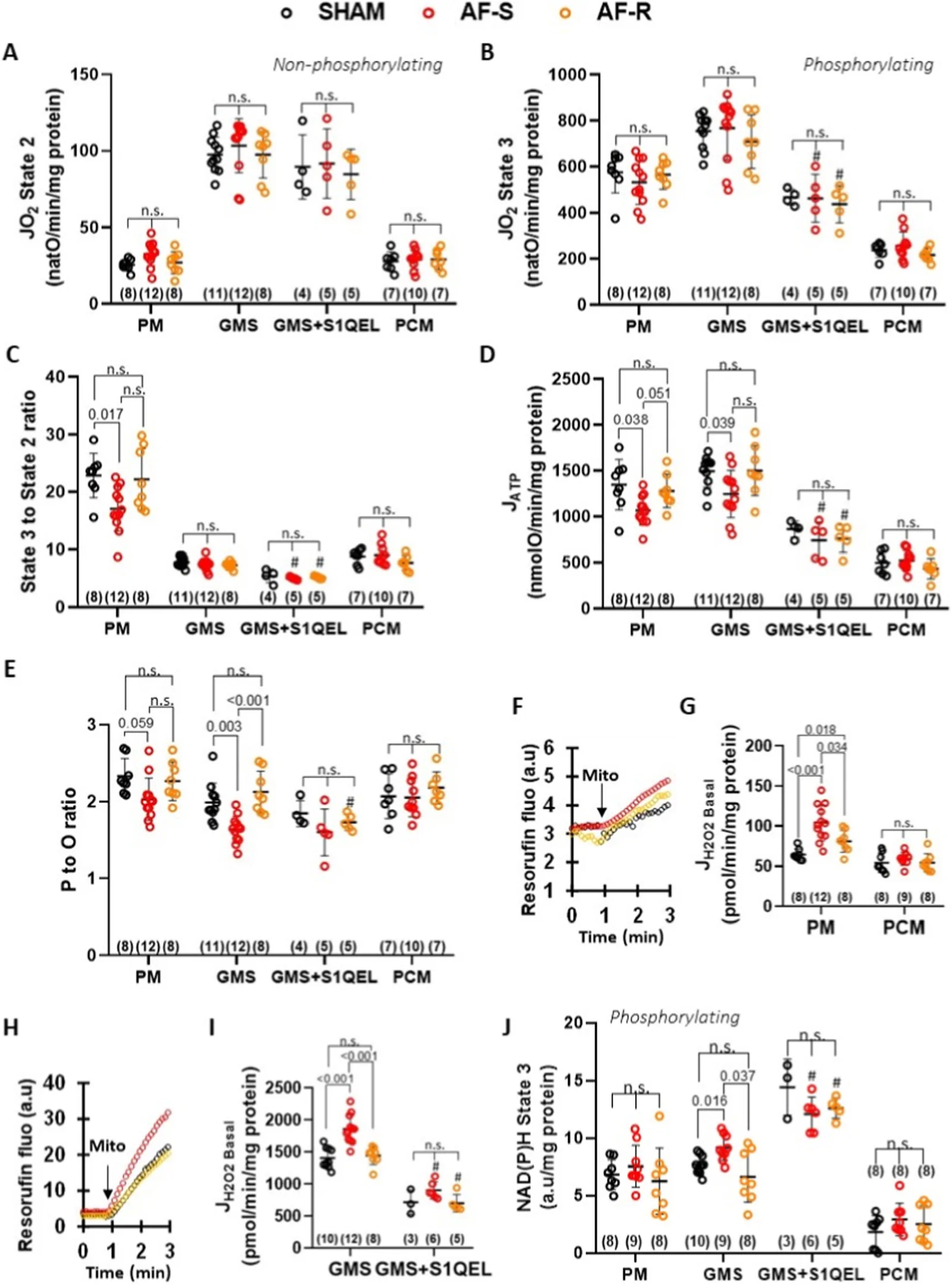
**O_2_-consumption, respiratory coupling, rate of ATP synthesis, and H_2_O_2_ emission from mitochondria**. Mitochondria were isolated and purified from LAA of Sham, AF-Sensitive (AF-S), and AF-Resistant (AF-R) sheep to evaluate (i) electron transport chain function, (panels **A:** State 2 non-phosphorylating respiration, **B:** respiration coupled to maximal ATP-synthesis (State 3, phosphorylating respiration) **C**: State 3 to State 2 ratio, **D**: rate of ATP synthesis, and **E:** P to O ratio reflecting OXPHOS efficiency. **F-I:** rate of hydrogen peroxide emission (H_2_O_2_ emission) assessed with pyruvate + malate or palmitoyl-carnitine + malate (PM or PCM, Panels **F** (raw traces) and **G**), or glutamate + malate + succinate in absence or presence of S1QEL (GMS or GMS + S1QEL, panels **H** (typical traces) and **I**). **J**: autofluorescence of the mitochondrial NAD(P)H pool obtained during state 3 respiration measurement (**B**). Panels show individual data points and mean ± SD. Statistical analysis was performed with *t*-test using the Holm-Sidak method to correct for multiple comparisons. The numbers in brackets represent individual experiments in each group. #*p* < 0.05 GMS + S1QEL *vs.* GMS, by Wilcoxon test for paired samples. Complementary analysis is presented in Supplemental Fig. 6.

AF-S sheep exhibited quantitatively (but not significantly) greater state 2 respiration under PM conditions (Fig. 4A), and reduced state 3 respiration (Fig. 4B) associated with a significantly decreased state 3 to state 2 ratio compared to Sham in the presence of PM (Fig. 4C). No differences were observed in other substrate combinations in state 2 or 3 respiration, nor between AF-R and Sham for any substrate condition (Fig. 4A–C). Isolated mitochondria from AF-S sheep showed a decreased ATP-synthesis rate with PM or GMS compared to Sham and AF-R, but not with PCM (Fig. 4D). The P to O ratio, representing oxidative phosphorylation (OXPHOS) efficiency, was significantly decreased in AF-S mitochondria with GMS compared to Sham and AF-R (with a similar trend in PM), but remained unchanged with PCM between the three groups (Fig. 4E). S1QEL decreased both state 3 respiration and ATP synthesis rate, and eliminated P to O differences observed in GMS among groups (Fig. 4E).

Mitochondrial ROS emission was significantly increased during state 2 respiration under PM conditions in AF-S and AF-R sheep, compared to Sham (Fig. 4F and G). This mitochondrial phenotype was not evident with PCM. In contrast, during succinate oxidation, only AF-S mitochondria showed increased ROS emission versus AF-R and Sham, which was prevented by S1QEL (Fig. 4H and I). Mitochondrial ROS emission during state 3 respiration was identical among groups (Supplemental Fig. 6A). Increased ROS emission in AF-S during succinate oxidation was associated with a more reduced NAD(P)H pool in state 3 respiration (Fig. 4J) when compared to Sham and AF-R and to Sham only during state 2 respiration (Supplemental Fig. 6B), indicating that redox metabolism is altered in AF-S mitochondria during succinate oxidation, independently of the bioenergetic state.

An increase in inner mitochondrial membrane polarization (ΔΨm) was observed in AF-S and AF-R compared to Sham under state 2 respiration in PM conditions (Supplemental Fig. 6C), which was only significant during ATP synthesis and after its inhibition by CAT in AF-R mitochondria. During succinate oxidation, AF-S mitochondria showed an apparent hyperpolarization compared to both Sham and AF-R (Supplemental Fig. 6D). The inner membrane hyperpolarization in AF-S sheep was significant versus Sham during state 3 respiration and after its inhibition by CAT. No differences among groups were observed with PCM (Supplemental Fig. 6E). Whilst a fixed matrix volume was used for all groups, the observed alteration in MFN1 expression, significantly increased in AF-S compared to Sham and AF-R (Supplemental Fig. 6F and Supplemental Table 4), suggests a potential change in mitochondrial morphology that could alter our measurements [36]. Note that although ΔΨm values depends on matrix volume (Supplemental Fig. 6G), any mitochondrial morphological change induced by changes in MFN1 could not on its own explain the 15-mV hyperpolarization observed in the AF-S group.

Since ΔΨm is the main driving force allowing calcium accumulation within the matrix, we measured the mitochondrial calcium uptake rate and maximal quantity of calcium needed to open the mitochondrial permeability transition pore (mPTP) during succinate oxidation. AF-S mitochondria exhibited a significant increase in calcium uptake compared to both Sham and AF-R (Supplemental Fig. 7A and B). Compared to Sham, mPTP sensitivity to calcium was increased in AF-R, and AF-S showed an intermediate phenotype (NS vs Sham or AF-R Supplemental Fig. 7C).

Thus, in-depth mitochondrial function analysis revealed common characteristics between AF-S and AF-R during PM oxidation, while several mitochondrial functions were only altered in AF-S during succinate oxidation, suggesting a specific mitochondrial dysfunction during succinate oxidation which occurs in the LAA of the AF-S group only (Fig. 3).

**Fig. 5:**
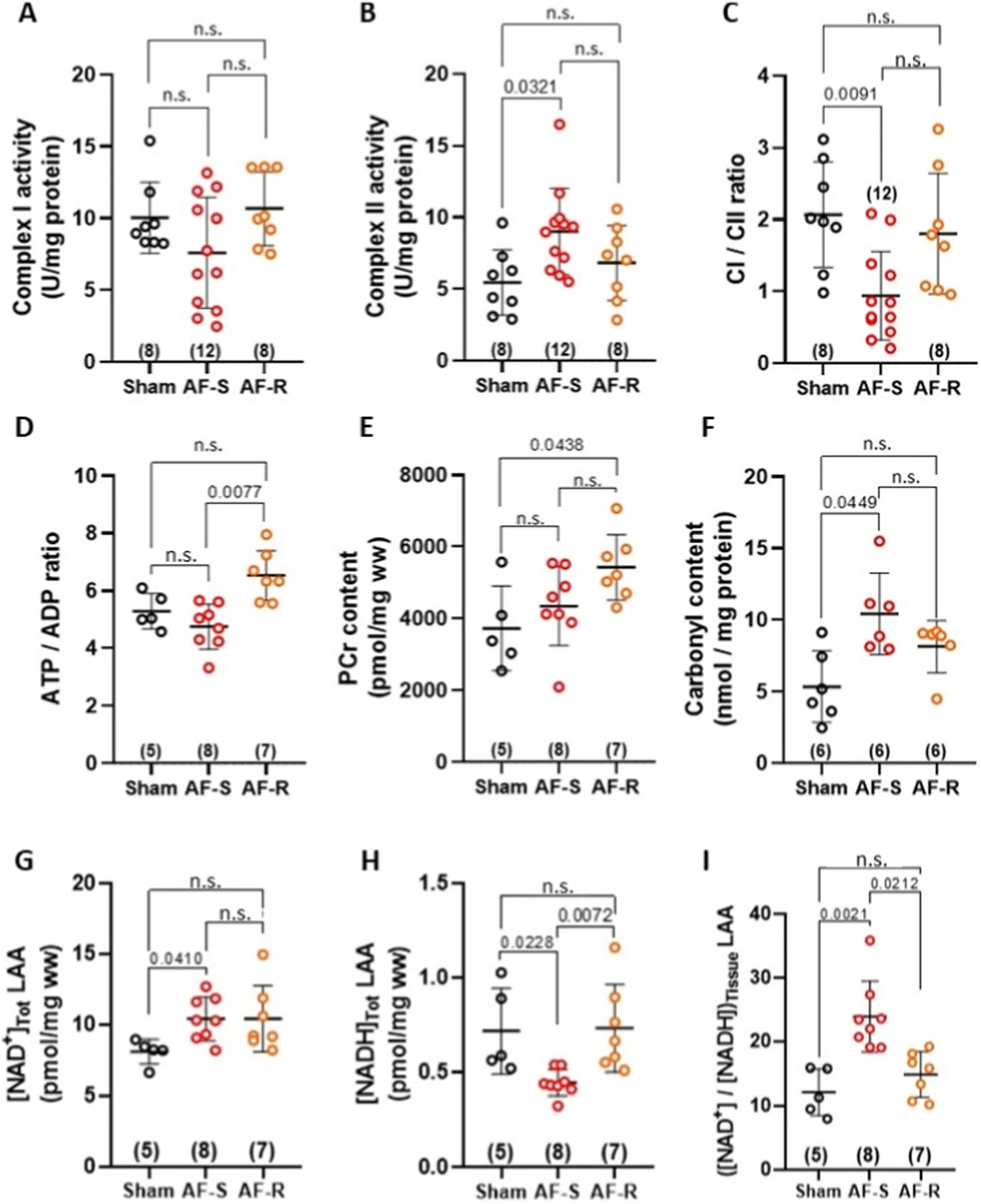
**Complexes I and II specific activity, and LAA energetic and redox status**. **A - C:** Mitochondrial complex I (**A**) and complex II (**B**) specific activity, and complex I/II activity ratio (**C**) studied in isolated mitochondrial from Sham, AF-S, and AF-R. **D** - **I**. ATP to ADP ratio (**D**), PCr content (**E**), Total carbonyl content (F), Total NAD^+^ (**G**), Total NADH (**H**), and NAD to NADH ratio (I) were studied in the LAA of the three groups by HPIC (see methods). Panels show individual data-points and mean ± SD. Statistical analysis in panels A to D, was performed with t-tests using the Holm-Sidak method for multiple comparisons. Statistical analysis in panels E to I, was performed by Kruskal-Wallis test and Dunn's post-hoc test for multiple comparisons. The numbers in brackets represent individual experiments.

The electron transport chain complex II (succinate oxidation) and complex I (NADH oxidation) competes for ubiquinone reduction, and as a consequence, can modulate the intramitochondrial level of NADH oxidation and ROS production. Thus, activity of complexes I and II was investigated on isolated mitochondria. No differences were observed at the level of complex I (Fig. 5A), while complex II activity was significantly increased in AF-S versus Sham (Fig. 5B–NS for AF-R versus Sham). The complex I to complex II activity ratio decreased in AF-S compared to Sham (Fig. 5C, AF-R N.S. vs Sham), consistent with a contribution of succinate to ROS emission (Fig. 4I), and with observed alterations in the autofluorescence of the NAD(P)H pool during succinate oxidation (Fig. 4J).

### 3.4 LAA energetic and redox status

Knowing that succinate accumulates within LAA of AF-S, and that *in vitro* mitochondrial P to O ratio decreased and ROS emission increased during succinate oxidation, at least in part secondary to CI to CII ratio decrease, we then assessed the LAA tissue energetic and redox status. PCr, ATP, ADP and AMP contents in LAA were quantified (Fig. 5 and Supplemental Fig. 8). AF-S LAA, compared to AF-R and Sham, showed no significant differences in AMP, ADP or ATP content, as well as ATP to AMP ratio or PCr to ATP ratio (Supplemental Fig. 8). In contrast, the ATP to ADP ratio was lower in AF-S compared to AF-R (Fig. 5D). Compared to Sham, AF-R LAA showed an increase in PCr content (*p* < 0.05, Fig. 5E), and a nearly significant increase in ATP content (*p* = 0.0562, Supplemental Fig. 8C). These changes were paralleled by a significant increase in the mitochondrial creatine kinase expression level (CKMT2, Supplemental Fig. 8F ), although we did not observe changes in total creatine kinase (CK) activity within LAA homogenates (Supplemental Fig. 8G).

The previously-mentioned results showing ROS emission increase during succinate oxidation in AF-S mitochondria led us to evaluate the extent of oxidatively-modified protein accumulation in the atrial compartment, as indicated by carbonyl content. Carbonyl content increased significantly in AF-S versus Sham, with no significant difference between AF-R and Sham (Fig. 5F).

Oxidative damage depends on the balance between ROS production and scavenging, which is partly controlled by the NADH to NAD^+^ ratio. Thus, to better understand the differential redox regulation within the atria of AF-S and AF-R sheep, NAD^+^ and NADH (cytosolic + mitochondrial) content were quantified. Compared to Sham, AF-S and AF-R mitochondria showed increased NAD^+^ total content (Fig. 5G), but AF-S LAA had significantly decreased NADH total content compared to both Sham and AF-R (Fig. 5H). Thus, the NAD^+^ to NADH ratio was significantly increased in AF-S LAA versus both AF-R and Sham, indicating a redox disturbance not observed in AF-R LAA (Fig. 5I), that would be expected to promote the accumulation of oxidative damage in AF-S.

**Fig. 6:**
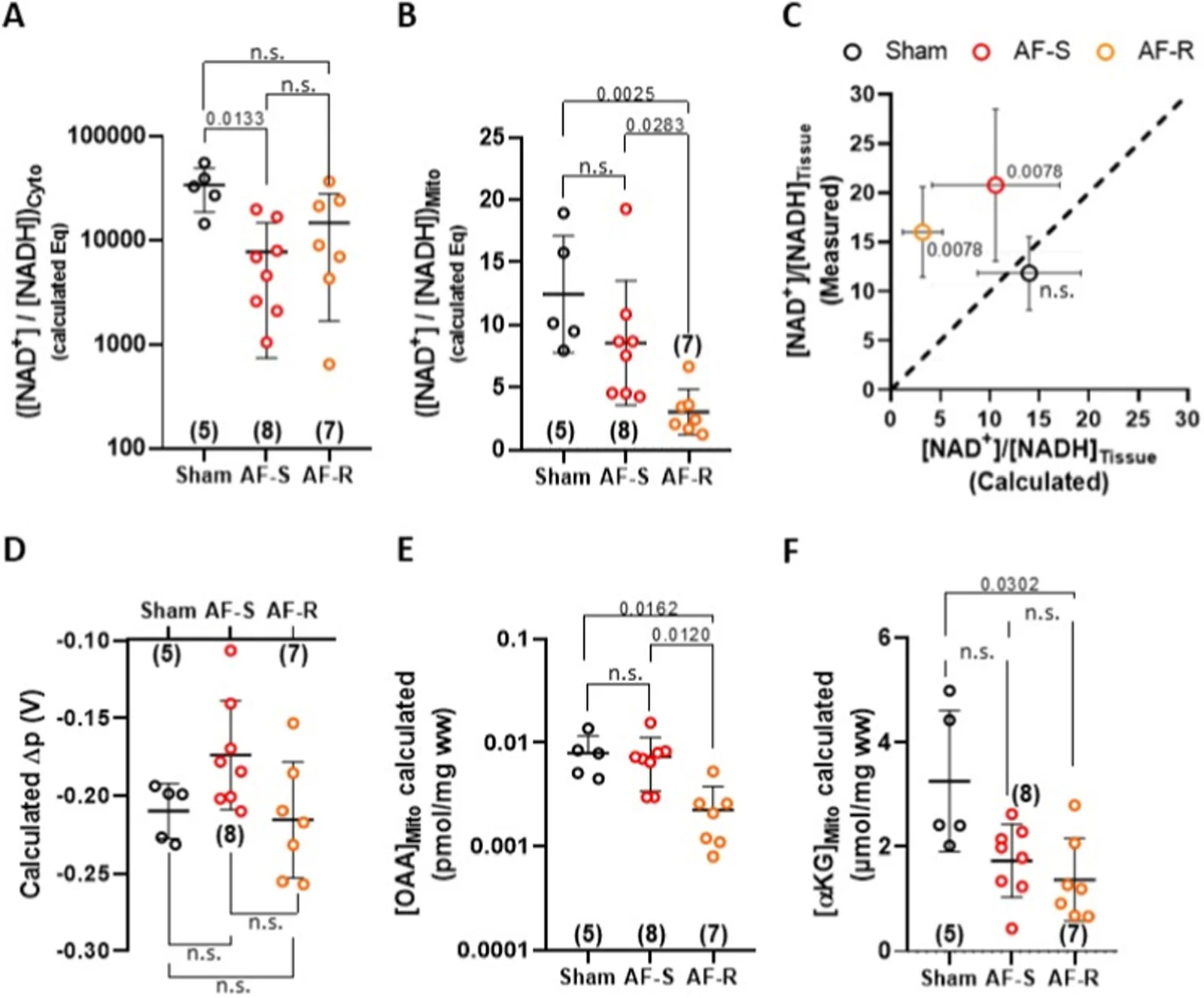
**Differential mitochondrial redox regulation within AF-R and AF-S LAA**. **A** and **B:** cytosolic (**A**) and mitochondrial (**B**) NAD^+^ to NADH ratio calculated using the l-lactate to pyruvate and β-hydroxybutyrate to acetoacetate ratio under thermodynamic equilibrium postulate for the reaction catalyzed by lactate dehydrogenase and β-hydroxybutyrate dehydrogenase. **C**. Graph representing the mean measured LAA tissue NAD ^+^ to NADH ratio as a function of the mean calculated NAD **^+^** to NADH ratio. **D:** calculated mitochondrial proton motive force (Δp) within the LAA tissue while considering the system at thermodynamic equilibrium. **E** and **F:** calculated mitochondrial oxaloacetate (**E**, [OAA]) and *α*-ketoglutarate (**F**, [*α*-KG]) concentrations. Panels show individual data-points and mean ± SD. Statistical analysis was performed by Kruskal-Wallis test and Dunn's post-hoc test for multiple comparisons. The numbers in brackets represent individual experiments.

To assess the differential redox regulation in the cytosol and mitochondrial compartments, which cannot be directly evaluated experimentally, we combined a metabolomic approach with simulations assuming that the systems studied were at thermodynamic equilibrium. Using the measured pyruvate to l-lactate ratio (Fig. 2E) and the l-lactate dehydrogenase equilibrium constant (Supplemental Material), the cytosolic [NAD^+^] to [NADH] ratio was calculated and suggested a more oxidized cytosolic compartment in AF-S compared to Sham LAA (Fig. 6A). The same approach was used to calculate the intramitochondrial [NAD^+^] to [NADH] ratio of the three groups. For this purpose, the LAA β-hydroxybutyrate to acetoacetate ratio was quantified in LAA (Supplemental Fig. 9A–C). Interestingly, AF-R LAA showed an increased matrix ratio of these ketone bodies (Supplemental Fig. 9C) compared to both Sham and AF-S. As a consequence, the calculated mitochondrial [NAD^+^] to [NADH] ratio is lower (reduced environment) in AF-R versus both Sham and AF-S (Fig. 6B). Furthermore, from these local redox ratios, the overall tissue NAD^+^ to NADH ratio at equilibrium was calculated considering the NAD(H)^+^ content in the cytosolic and mitochondrial compartments (N_ratio_) as well as the ratio of the volumes of the two compartments (V_ratio_). Plotting the measured [NAD^+^] to [NADH] ratio as a function of the calculated value (Fig. 6C), showed that in Sham LAA there was no significant difference (Wilcoxon rank sum test) between the measured and calculated ratio. Thus, the redox system of Sham LAA works close to equilibrium. In contrast, the AF-S and AF-R groups are clearly far from the equilibrium hypothesis (*p* = 0.0078 for each), possibly an effect secondary to metabolic remodeling.

Owing to the fact that the calculated overall redox ratio of the tissue is mainly fixed by the mitochondrial NAD^+^ to NADH ratio (Supplemental Fig. 9D), it is possible that β-HBDH may be working far from thermodynamic equilibrium in AF-R LAA and to some extent, in AF-S LAA, compared to Sham. Importantly, we can then argue that the increased [NAD^+^] to [NADH] ratio, experimentally measured within LAA tissue of the AF-S sheep, reflects oxidation of the mitochondrial compartment compared to Sham and AF-R. In this context, the calculated mitochondrial proton motive force (Δp) shows that AF-S mitochondria tend to have a lower Δp compared to AF-R (Fig. 6D). Thus, AF-S mitochondria *in vivo* could have a higher rate of oxygen consumption compared to AF-R, which is in line with the previously observed NAD^+^ oxidation state. This approach was also used to calculate the relative [oxaloacetate] ([OAA]), [alpha-ketoglutarate] ([*α*-KG]) and [l-malate] content within the cytosolic and mitochondrial compartments (Fig. 6E and F) and Supplemental Fig. 9E–G). AF-R mitochondria showed a lower [OAA]_mito_ compared to Sham and AF-S (Fig. 6E) and a lower [*α*-KG]_mito_ (Fig. 6F) than Sham, further suggesting that AF-R LAA have a differential remodeling of the TCA cycle versus AF-S.

Taken together, our results suggest that in our ovine model, AF stabilization is paralleled by TCA cycle remodeling, mitochondrial succinate accumulation and oxidation of the mitochondrial compartment. Based on the data in Figs. 3, 4E and 4I, 5F, 5H, and 5I, we hypothesized that succinate accumulation participates in metabolic disturbances that stabilize AF in AF-S sheep.

### 3.5 Succinate and atrial arrhythmia susceptibility in a rodent model

**Fig. 7:**
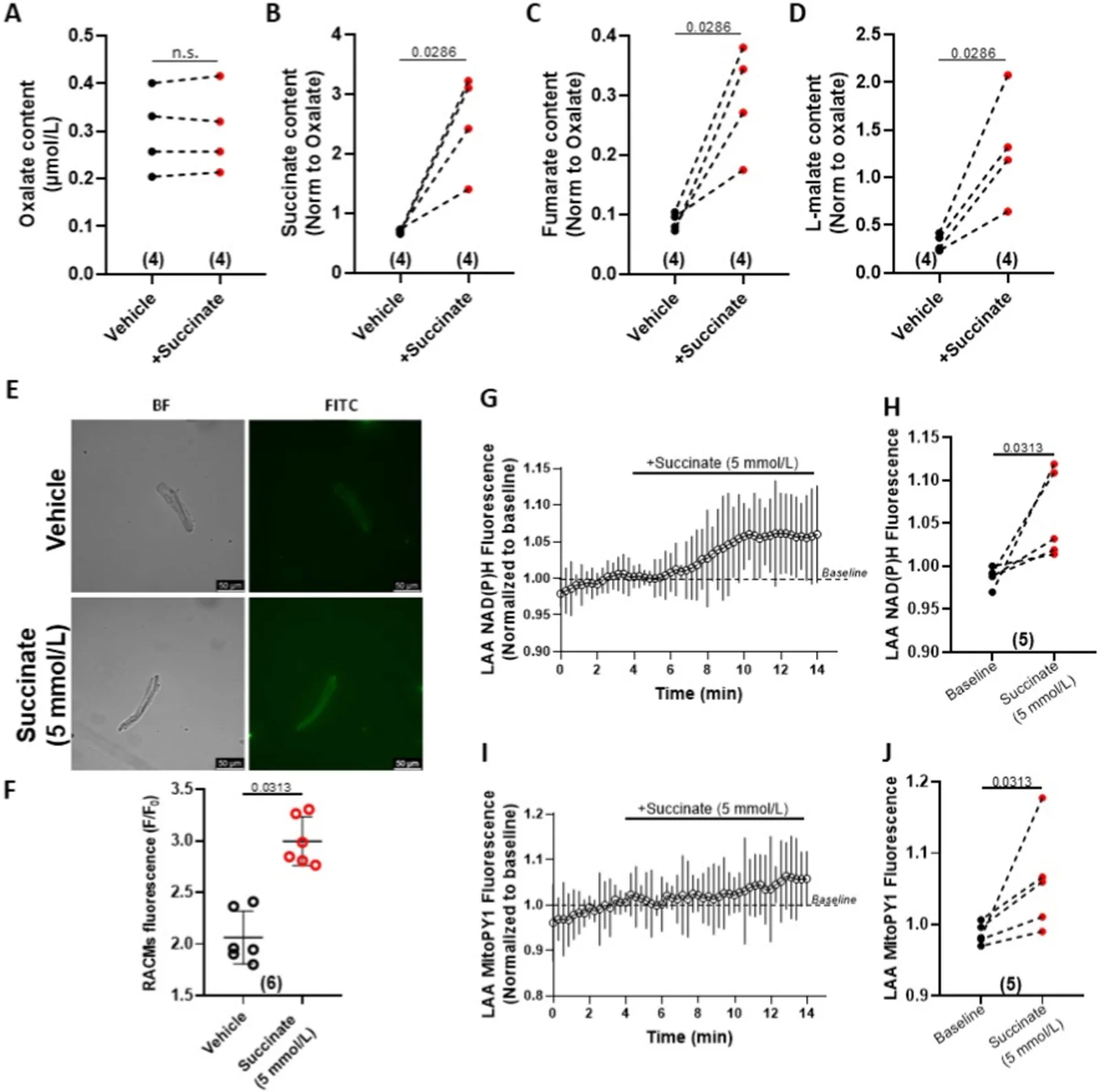
**Effect of exogenous succinate application on atrial cardiomyocytes**. **A-D:** Isolated rat atrial cardiomyocytes (RACMs) were incubated with 5 mmol/L of succinate and intracellular metabolites content was assessed by mass spectrometry. Signals correction-quantification and normalization were performed using both deuterium-labelled succinic acid and oxalic acid (**A**), respectively (see methods). Intracellular content of succinate (**B**), fumarate (**C**) and l-malate (**D**) is represented. **E:** Representative images illustrating ROS emission measurement by CellROX Green in RACM treated with either succinate at 5 mmol/L or vehicle (BF: bright field, FITC: Fluorescein). **F:** Quantification of intracellular ROS emission in presence of succinate measured with CellROX, with respect to the background. **G-J:** Evolution of LAA NAD(P)H (**G** (time course), **H** (quantification)) and MitoPY1 (H_2_O_2_ sensitive fluorescent probe, **I** (time course), **J** (quantification)) fluorescence measured in the *ex vivo* perfused rat heart, using an optical fiber positioned close to the LAA, upon perfusion with 5 mmol/L succinate. **A-D:** Statistical analysis *via* Mann-Whitney, N = 4 in each group Test. **F, H and I:** Statistical analysis *via* Wilcoxon test for paired samples, N = 6 panel F and N = 5 panels G to J. Complementary analysis is presented in Supplemental Fig. 10.

To assess the potential role of succinate accumulation in AF, we first evaluated whether acute exposure of rat atrial cardiomyocytes (RACMs) to exogenous succinate can alter atrial metabolism, ROS emission and NAD(P)H fluorescence *ex vivo* in beating rat hearts and *in vitro* in RACMs. Succinate is a dicarboxylate, known (at least in ventricular cardiomyocytes or skeletal muscle) to be transported across the membrane only in acidic conditions (*i.e.* ischemia, hypoxia or intense exercise) [37,38]. RACMs were incubated with a high concentration of succinate (5 mmol/L) and its impact on TCA cycle metabolites level assessed by mass spectrometry. Following succinate incubation, the intracellular content of different mitochondrial TCA cycle and glycolytic metabolites was assessed by mass spectrometry using deuterium-labelled succinate (see Methods) and internal oxalate content (Fig. 7A) used for signal correction and normalization. This analysis showed that the intracellular concentrations of succinate, fumarate and l-malate were increased with succinate exposure, while citrate and alpha-ketoglutarate showed a trend towards increase (Fig. 7B to D and Supplemental Fig. 10A and B). Three glycolytic intermediates (glucose-6-phosphate, fructose-1,6-bisphosphate and pyruvate) showed no changes following RACMs incubation with exogenous succinate (Supplemental Fig. 10C–E). Incubation of RACMs with exogenous succinate significantly increased the intracellular succinate concentration by 3.8 fold (Supplemental Fig. 10F). At the functional level, RACMs incubation with succinate significantly increased ROS emission (Fig. 7E and F, and Supplemental Fig. 10G), and succinate perfusion of Langendorff-perfused beating rat hearts significantly increased both NAD(P)H and MitoPY1 LAA fluorescence (Fig. 7G–J). H_2_O_2_ perfusion showed no impact on NAD(P)H fluorescence and produced a net increase in MitoPY1 fluorescence (Supplemental Fig. 10H–K).

**Fig. 8:**
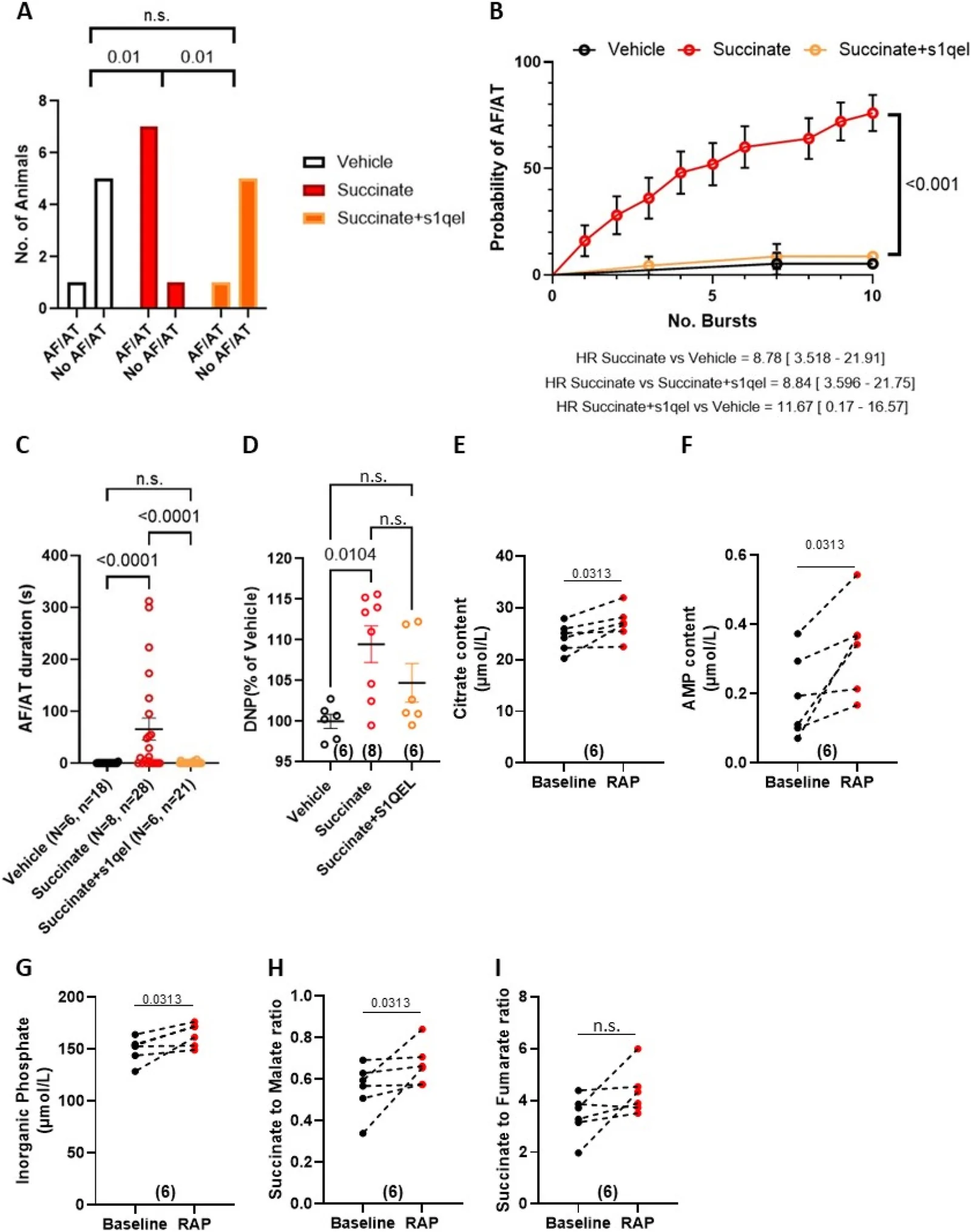
**Effect of succinate on arrhythmia susceptibility and carbonylation *in vivo* and effect of RAP on blood metabolites**. Atrial arrhythmia susceptibility was assessed *in vivo* by transesophageal burst-pacing following intravenous injection of vehicle, succinate or succinate + S1QEL. **A:** Number of animals with inducible AF/AT versus no arrhythmia. **B:** Cumulative probability of AF/AT as function of number of induction-bursts. **C:** AF/AT duration in response to a burst-pacing challenge. **D:** Carbonyl content of *in vivo* hearts measured by Oxyblot. **E-I:** Blood was harvested before (baseline) or after *in vivo* atrial tachystimulation by transesophageal burst-pacing (RAP), and citrate (**E**), adenosine monophosphate (AMP, **F**), inorganic phosphate (**G**) were assessed by mass spectrometry (N = 6). **H**: Succinate to l-malate ratio. **I**: Succinate to fumarate ratio. Statistical analysis by Fischer exact test (**A**), Log-rank Mantel-Cox test (**B**), Kruskal-Wallis test and Dunn's post-hoc test for multiple comparisons (**C, D**), and Wilcoxon test (**E**-**I**). Complementary analysis is presented in Supplementary Fig. 11.

*In vivo* intravenous succinate administration increased atrial tachyarrhythmia vulnerability, susceptibility, and duration compared to values in both vehicle-infused and S1QEL pretreated animals (Fig. 8A–C). Succinate-injected animals showed a significant increase in carbonyl content (Fig. 8D, and Supplemental Fig. 11A and B). Succinate + S1QEL-treated rats had carbonyl content not significantly different from vehicle, but the reduction versus succinate-only was not statistically significant (Fig. 8D).

In order to further explore the blood metabolic changes caused by acute atrial tachypacing *in vivo*, serum from rats subjected to RAP was analyzed by mass spectrometry. Acute atrial tachyarrhythmia was associated with the development of a specific blood metabolic profile compared to paired baseline control. RAP increased citrate, AMP, and inorganic phosphate, and caused a trend towards cis-aconitate increase, reflecting mitochondrial metabolic activation and increased energy consumption (Fig. 8E–G, and Supplemental Fig. 11C). A significant increase of the succinate to l-malate ratio and a trend towards increased succinate to fumarate ratio were observed (Fig. 8H and I), although succinate, fumarate and l-malate were not changed (Supplemental Fig. 11D–F).

## 4 Discussion

### 4.1 Major findings and novelty

We investigated the mechanisms underlying AF sensitivity and resistance in a sheep model, revealing the involvement of metabolic, bioenergetic, redox and mitochondrial function differences. Notable findings in AF-S animals, compared to AF-R or Sham, included: i) altered proteomic, enzymatic and metabolomic profiles involving glycolysis, fatty acids utilization, and TCA cycle function/regulation; ii) increased succinate content, decreased ATP/ADP ratio, and NADH/NAD^+^ ratio at the tissue level, iii) increased activity of PDH and *α*-KGDH, paralleled by decreased fumarase activity likely participating to succinate tissue accumulation, iv) increased succinate-driven ROS emission; v) decreased OXPHOS efficiency during succinate oxidation, vi) altered CI/CII activity ratio, and vii) increased mitochondrial [NAD^+^]/[NADH] ratio reflecting intramitochondrial oxidation. Using the rat model, we show that atrial cardiomyocytes can metabolized exogenous succinate, and that this is paralleled by a redox imbalance and an increased probability/stability of tachypacing-induced atrial arrhythmias. Moreover, we show that atrial tachypacing alone in the rodent leads to the development of a specific blood metabolic signature including an increased succinate to l-malate ratio.

Finally, our analysis combining metabolite content assessment on *in vivo* freeze-clamped sheep LAA tissues with metabolic simulation placing the system at the thermodynamic equilibrium suggests that resistant animal have a higher intramitochondrial NADH to NAD^+^ ratio than AF-S sheep. This profile would be adaptative to the massive energetic burden caused by RAP, in terms of both OXPHOS efficiency and ROS detoxification capacity when compared to the profile in AF-S sheep:

• Firstly, increased matrix NADH to NAD^+^ ratio would promote electron fueling to complex I (push regulation), potentially improving the energetic status of AF-R atria compared to AF-S. Moreover, AF-R animals showed improved ketone body metabolism reflected by the increased β-OHB to AcAc (acetoacetate) ratio which might improve intramitochondrial NADH production.
• Secondly, a higher mitochondrial NADH level should favor intramitochondrial NADPH and GSH production, thus improving ROS scavenging and protecting mitochondrial function from oxidative damages.

Moreover, AF-R LAA showed adaptation leading to an optimization of PCr production which should further participate to buffer the increased energetic demand RAP-induced. In contrast, the decreased intramitochondrial NADH to NAD^+^ ratio in AF-S mitochondria would further favor succinate-supported respiration over NADH-linked respiration. This would decrease the proton-pumping contribution of complex I to maintain proton gradient across the inner membrane and consequently promote a decrease of OXPHOS efficiency. This phenomenon combined with a decreased AK expression level (Supplemental Table 4) should strongly contribute to altered energetic fluxes between mitochondria and the contractile apparatus.

From an electrophysiological standpoint, our model showed that reduction of atrial refractoriness is not sufficient to explain sensitivity to persistently domesticate AF. AF-S animals did show a longer P-wave duration before the start of the RAP protocol, and they presented reduced atrial conduction velocities and reduced Cx40 mRNA and protein content compared to AF-R but not to Sham. P-wave duration >120 ms is well associated with the clinical risk of incident AF [39] and Cx40 reduction in atrial tissue has been reported as a feature of human chronic AF [40]. Collagen deposition with consequent endomysial fibrosis could also partly explain the changes in atrial conduction velocity, and this has been associated in patients with increased risk of AF recurrence [41]. Our model thus recapitulates electrophysiological risk factors of new-onset AF and presents electrical and structural changes similar to those encountered in chronic AF patients. Taken together our data further suggest that metabolic remodeling should be considered as an AF substrate [42].

**Fig. 9:**
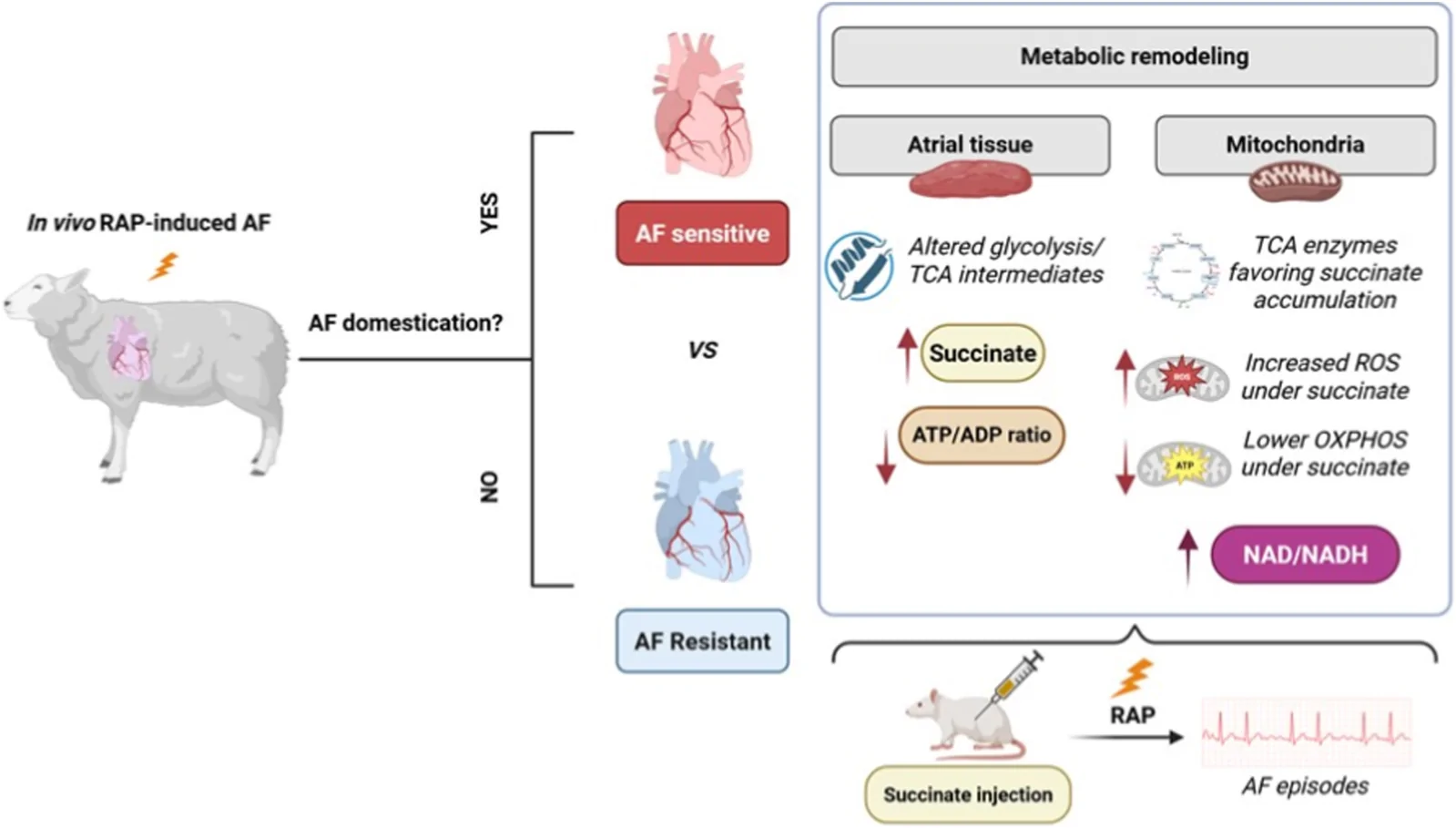
**Scheme summarizing the study findings**. Electrically-maintained AF led to domestication in AF-sensitive (AF-S) animals within 3 weeks, but some animals were AF resistant (AF-R) and failed to develop self-sustained AF despite 4 months of electrically maintained AF. Specific patterns of mitochondrial and metabolic remodeling characterized AF-S. In the presence of succinate, AF-S mitochondria showed increased ROS emission, decreased P to O ratio, increased complex II to complex I activity ratio. AF-R and AF-S mitochondria showed differential regulation of the TCA cycle. At the whole-tissue level, AF-S atria showed more oxidized proteins compared to Sham, along with an increased NAD^+^ to NADH ratio and impaired energy availability reflected by a decreased ATP to ADP ratio relative to both Sham and AF-R values. AF-R LAA showed a higher PCr paralleled by increased CKMT2 expression and a more reduced mitochondrial matrix secondary to increased ketone body metabolism compared to AF-S. AF-S also showed differential TCA cycle enzymes regulation compared to AF-R, likely contributing to succinate accumulation in the AF-S group… (Created with BioRender.com.)

This is the first study, to our knowledge, to explore and identify metabolic factors differentiating AF-R animals from AF-S in a clinically relevant large animal model, highlighting the critical role of TCA cycle regulation and mitochondrial redox state in contributing to AF resistance. A schematic summary of the main findings of the study is provided in Fig. 9.

### 4.2 Involvement of succinate buildup, redox impairment, and TCA cycle remodeling in AF

Our data suggest that AF-S animals showed LA succinate accumulation secondary to TCA cycle remodeling leading to (i) a two-step activation before complex II (PDH and *α*-KGDH) and (ii) a two-step inhibition after complex II (Fumarase and MDH2). Indeed, combined analysis of TCA cycle metabolites content and mitochondrial enzyme activities suggests that increased mitochondrial pyruvate supply and transport (increased pyruvate to l-lactate ratio), enhanced acetyl-CoA generation (increased PDH activity), and increased α-KGDH activity, together with reduced fumarase and MDH2 activities, would favor succinate accumulation in AF-S sheep atria. Moreover, this profile was associated with a decreased CI to CII activity ratio, favoring further succinate oxidation by complex II towards NADH oxidation by complex I. In contrast, AF-R mitochondria exhibited a distinct metabolic remodeling profile characterized by a significantly reduced SCS that could limit succinate production, and may contribute to the accumulation of isocitrate relative to AF-S. AF-S isolated mitochondria showed altered energetic output, increased ROS emission, and redox imbalance during succinate oxidation. Importantly, to get insights into the mitochondrial energy metabolism within the tissue, mitochondrial NAD^+^ to NADH ratio as well as Δp were calculated with the postulate of a system working close to the thermodynamic equilibrium, *i.e.* metabolic fluxes are rather small compared to enzyme capacities. As shown in Fig. 6C, this hypothesis fully describes the physiological situation in the Sham group, while the AF-R group is more displaced from the equilibrium hypothesis than AF-S. Considering this analysis and the evolution of Δp in the LA, we hypothesize that AF-S mitochondria in the LA are more depolarized than Sham or AF-R mitochondria. This is important when trying to extrapolate the functional impact of succinate accumulation on mitochondrial ROS emission because the lower the Δp the lower the succinate-driven ROS emission. In this context, our calculations suggest that AF-S mitochondria would be more depolarized than AF-R mitochondria, and thus we cannot claim on the mechanism linking succinate accumulation and mitochondrial ROS emission. Nonetheless, our results regarding carbonylation and the redox state of AF-S LA tissue indicate that the system is strongly oxidized in AF-S sheep. Thus, oxidative damage in AF-S LA tissue might be a consequence of both succinate buildup and failure of the ROS scavenging system secondary to a decreased intramitochondrial NAD(P)H to NAD(P)^+^ ratio [43].

To our knowledge, this is the first report of a specific atrial accumulation of succinate and linked mitochondrial redox alteration in a clinically relevant animal model. Succinate serves as a substrate for complex II in the respiratory chain and operates as a signaling molecule through the SUCNR1 receptor (formerly GPR91) [44,45]. Its accumulation has been implicated in various cardiovascular diseases [15,45,46], but its role remains relatively unexplored in AF, although a recent report showed that chronic systemic succinate overload in mice was mechanistically linked to atrial arrhythmias [17]. Moreover, elevated plasma succinate levels were associated with atrial dysfunction, stroke recurrence [15], and AF [16]. Succinate also accumulates in the ischemic heart [47], and AF has been suggested to be associated with supply-demand imbalance ischemia [48]. Examining the expression of both HIF1*α* and MCT4 (downstream to HIF1*α*) by proteomic analysis we did not observe strong sign of hypoxia (https://doi.org/10.6084/m9.figshare.28269872.v1). Thus, in the fibrillating sheep atria, succinate accumulation does not seem to be secondary to hypoxia; direct measurement of tissue oxygen tension and myoglobin redox state might be helpful in addressing this issue.

AF-risk development in patients has been positively correlated with accumulation of D/L-2-hydroxyglutarate in the blood [16]. In the sheep model this metabolite does not seem to play a role in AF stabilization. Future experiments evaluating the time course of both TCA cycle remodeling and succinate buildup during AF progression, in relation to electrophysiological abnormalities, would help to decipher if and how succinate accumulation and mitochondrial redox alteration participate in AF progression and/or stabilization. It would also be necessary to compare glycolytic and TCA fluxes between AF-R and AF-S atria in order to precisely evaluate the redox state of the mitochondrial compartment between AF sensitive and resistant animals.

### 4.3 Involvement of LA energetic status and substrate metabolism in AF resistance

Increased energy demand caused by AF has different consequences in AF-S versus AF-R sheep. AF-R sheep showed (versus AF-S) greater ATP to ADP ratio (Fig. 5D), increased mitochondrial content (Supplemental Fig. 5C) and larger PCr content than Sham (Fig. 5E), with increased mitochondrial CK content (CKMT2 https://doi.org/10.6084/m9.figshare.28269872.v1, Supplemental Fig. 8F and Supplementary Table 4), suggesting that AF-R mitochondria are optimized to produce energy and export it towards the cytosolic compartment. Such metabolic adaptations in AF-R LAA might contribute to AF-resistance. AF-S LAA showed specific mitochondrial function disturbances not observed in AF-R, particularly during succinate oxidation (Fig. 4, Fig. 5). Moreover, AF-S atria showed a decreased content of AK, which is a critical component of the creatine kinase shuttle system essential to cardiac energy homeostasis [49]. The relationship between atrial energetic status and AF stabilization is likely underscored in our study because we have not analyzed the local ATP to ADP ratio, adenine nucleotides compartmentation, and energy fluxes between mitochondria and myosin heads, which are very likely strongly disturbed in AF-S secondary to AK deficiency [50]**.**

Atrial energy metabolism is compromised by AF in patients and experimental models [8,19,[51], [52], [53], [54]], characterized by decreased expression of assembling factors or core proteins of complexes I and II, reduced activity of these complexes [8,9,55], oxidative damage, and increased mitochondrial ROS production [8]. A recent study suggests that increased cardiomyocyte mitochondrial content and metabolic function are linked to AF-stabilization [19]. Prior studies also implicated mitochondrial dysfunction and energetic deficiencies in AF [52,[56], [57], [58], [59]]. Our study goes beyond this previous work by evaluating metabolic changes associated with AF-resistance in an *in vivo* model and by identifying a potential key role of succinate accumulation in AF-sensitivity.

Reduced mitochondrial OXPHOS efficiency in AF-S mitochondria was observed with succinate only, but not with either fatty acid substrates (like palmitoyl-carnitine) or complex I linked respiratory substrate. Like previously reported in AF [60], our data suggest a shift towards glucose utilization in AF-S through increased (i) HK activity, (ii) pyruvate content, (iii) pyruvate to l-lactate ratio, and (iv) a lower cytosolic NAD^+^ to NADH ratio reflecting putative glycolysis activation, although atrial cardiomyocytes glucose consumption must be measured to confirm such statement. Moreover, both AF-S and AF-R groups showed increased atrial G6P and glycogen content, while l-lactate content or LDH activity were unchanged.

### 4.4 The potential role of succinate accumulation and oxidation

In mouse permeabilized cardiac mitochondria, succinate oxidation by complex II is favored over NADH oxidation by complex I [61]. This phenomenon could explain the impaired NADH oxidation by complex I (Fig. 4J and Supplemental Fig. 6B ), decreased OXPHOS efficiency (Fig. 4E), and increased ROS emission (Fig. 4I) observed in AF-S isolated mitochondria during succinate oxidation. Increased succinate in the matrix of AF-S mitochondria would be expected to participate to the reduced OXPHOS efficiency we observed under complex I bypass conditions. The competition between mitochondrial CI and CII to fuel the ETC is regulated by oxaloacetate [61], an endogenous natural metabolic inhibitor of complex II [62]. The calculated [OAA]_Mito_ were equivalent in both Sham and AF-S groups, thus in the AF-S group metabolic competition would not decrease succinate oxidation at the level of complex II.

A known effect of succinate accumulation is the stimulation of ROS overproduction *via* complex I [35], although global ROS emission is directly dependent on the Δp which experimentally we maintained at a high level (i.e. absence of ATP-synthesis), to observe maximal succinate-driven ROS emission. In the fibrillating atria ADP is available and consequently such bioenergetic conditions cannot exist. Thus, in the fibrillating atria of AF-S sheep, it is unlikely that succinate accumulation promotes increased ROS emission by reverse-electron transfer mechanism [63]. Moreover, the AF-S atrial tissue is oxidized and the calculated Δp tended to be lower than in Sham and AF-R atria, suggesting a higher rate of oxygen consumption in AF-S. Other sites within the respiratory chain can lead to ROS emission [64], and our data show that ROS emission in AF-S mitochondria is also increased compared to AF-R in absence of succinate (Fig. 4F and G). Thus, at this stage the mechanism promoting ROS emission in the self-sustained AF atria is still to be elucidated.

In patients, the principal source of ROS emission shifts from the cytosol to mitochondrial complex I during AF progression [11]. In that study [11], ROS emission was sensitive to rotenone, an inhibitor of complex I, which inhibits succinate-induced ROS emission in presence of a high Δp. We found that S1QEL, a specific inhibitor of succinate-induced ROS emission at the level of complex I [35], blocked succinate-induced ROS overproduction in AF-S under non-phosphorylating conditions (*i.e.* high Δp). In isolated mitochondria, during succinate oxidation, the mitochondrial inner membrane hyperpolarized and the NAD(P)H pool was more reduced, while *in vivo*, despite succinate accumulation the mitochondrial [NAD^+^] to [NADH] ratio was increased and Δp decreased.

In AF-S mitochondria, inner membrane hyperpolarization during succinate oxidation is paralleled by an increased rate of calcium uptake. Further studies are needed to examine the effects of enhanced mitochondrial ROS emission in AF-S animals on sarcoplasmic reticulum calcium handling, which is involved in promoting AF-susceptibility [65]. Nonetheless, several studies have shown that AF is associated with an increased calcium leak *via* RyR2 which will result in increased ATP consumption. AF-R atria showed adaptative changes, including a lower mitochondrial NAD^+^ to NADH ratio, that favor (i) electron fueling at complex I level, (ii) ROS detoxification and a higher expression level of mitochondrial creatine kinase that improve PCr output. These adaptations might contribute to AF resistance by allowing the preservation energy supplies and calcium homeostasis.

Exogenous application of succinate on RACMs increased (i) intracellular concentrations of succinate, fumarate and l-malate, (2) ROS emission and NAD(P)H fluorescence (like in isolated mitochondria) in the left atrial appendage of the perfused heart. Thus, rat atria can metabolize exogenous succinate. Exogenous succinate administration *in vivo* heightened AF susceptibility and stability. The mitigation of succinate's effects *in vivo* with S1QEL, along with the inhibitory effect of S1QEL on mitochondrial succinate-induced ROS emission, support the link between increased succinate, ROS emission and AF vulnerability. Succinate has been reported to show paracrine effects in different tissues [63,66,67], but no information is available about possible paracrine effects in the atria.

While we cannot exclude this possibility, the work reported here strongly suggests that TCA cycle remodeling, succinate overload, and tissue redox and energetic homeostasis changes contribute to AF domestication.

### 4.5 Potential limitations

The timing of bioenergetic remodeling and AF domestication should be studied to evaluate the causality between TCA cycle remodeling and AF domestication. Great caution should be taken when linking succinate buildup to mitochondrial ROS emission *in vivo*. Indeed, mitochondrial succinate-driven ROS emission is regulated by Δp, the value of which is not known *in vivo* in the fibrillating atria. Our findings regarding acute AF induction in rats provides direct support to the notion that succinate accumulation can promote AF susceptibility, but needs to be extrapolated cautiously to chronic AF in sheep or humans.

## 5 Conclusions

This is the first study to our knowledge to investigate the bioenergetic mechanisms underlying resistance to AF domestication in a large-animal model with electrical and structural similarities to clinical persistent AF. Our results suggest that disturbed mitochondrial succinate metabolism and altered mitochondrial redox balance may play an important role in sensitivity to AF domestication.

There appears to be a link between TCA cycle remodeling, succinate buildup, OXPHOS efficiency regulation, mitochondrial redox balance and AF progression. This work suggests that understanding the regulation of the TCA cycle during AF progression might help to develop new therapeutic strategies aiming to prevent AF progression in humans.

## CRediT authorship contribution statement

**Guido Caluori:** Writing – review & editing, Writing – original draft, Software, Methodology, Investigation, Formal analysis, Data curation. **Stanley Nattel:** Writing – review & editing, Writing – original draft, Methodology, Funding acquisition, Formal analysis. **Benoît Pinson:** Writing – review & editing, Methodology, Investigation, Formal analysis, Data curation. **Patrice Naud:** Methodology, Formal analysis, Data curation. **Bertrand Beauvoit:** Writing – review & editing, Methodology, Formal analysis. **Stéphane Claverol:** Methodology, Investigation, Formal analysis, Data curation. **Fanny Vaillant:** Writing – review & editing, Methodology, Investigation, Formal analysis. **Sabine Charron:** Methodology, Investigation, Formal analysis. **Florent Guilloteau:** Formal analysis, Data curation. **Virginie Loyer:** Resources, Methodology. **Marion Constantin:** Methodology, Investigation, Formal analysis. **Miruna Popa:** Methodology, Investigation. **Andrei Belykh:** Methodology, Investigation. **Anaïs Rogowsky:** Investigation. **Andreas Haeberlin:** Resources, Methodology. **Sylvain Ploux:** Methodology. **Hassan Adam Mahamat:** Software, Investigation. **Rémi Dubois:** Software, Investigation. **Bastien Guillot:** Methodology, Investigation. **Philipp Krisai:** Methodology, Investigation. **Tsukasa Kamakura:** Methodology, Investigation. **Olivier Bernus:** Methodology, Investigation. **Pierre Jaïs:** Project administration, Funding acquisition, Conceptualization. **Pierre Dos Santos:** Writing – review & editing, Writing – original draft, Supervision, Methodology, Funding acquisition, Conceptualization. **Philippe Pasdois:** Writing – review & editing, Writing – original draft, Validation, Supervision, Resources, Project administration, Methodology, Investigation, Funding acquisition, Formal analysis, Data curation, Conceptualization.

## Ethics declaration

This study was conducted in accordance with the following guidelines for animal welfare and/or reporting: Experiments followed Directive 2010/63/EU (European Parliament) on animal-use for scientific research. Protocols were approved by the local ethical committee (CEEA50) at University of Bordeaux and by the French Government (APAFIS#20364 and APAFIS#29528, for sheep and rat, respectively). This study was approved by the CEEA50 from University of Bordeaux. (Approval No. APAFIS#20364 and APAFIS#29528)

## Funding

This work was supported by the “10.13039/501100001665Agence Nationale de la Recherche (ANR-17-CE14-0029-01 (PJ) and ANR-10-IAHU-04)”, the Canadian Institutes of Health Research (SN), the 10.13039/100004411Heart and Stroke Foundation (SN), the Fondation Lefoulon-Delalande (GC), the Foundation Bordeaux University (GC), the 10.13039/501100003100French Federation of Cardiology (GC), and the 10.13039/501100001659Deutsche Forschungsgemeinschaft (10.13039/501100001659DFG, 10.13039/501100001659German Research Foundation) - project number 514892030 (MP). The Integrion ionic chromatography station was acquired with the financial support of SIRIC BRIO2 (COMMUCAN) and Region Nouvelle-Aquitaine (AAPPF2021-2020-12000110 to 10.13039/100004364BP). The ICS600 chromatography station (ICS6000) and the high-resolution mass spectrometer (Exploris 120) were acquired with financial support from the “Contrat de Projet État Région” 2021-2027 (Biomarker OMICS to 10.13039/100004364BP) and from the ITMO Cancer of Aviesan (I23007FS to 10.13039/100004364BP) as part of the 2021-2030 Cancer Control Strategy, with funds administered by INSERM.

## Declaration of competing interests

The study was conducted with Research Academic funds only. None of the Authors have relationships with the industry relevant to the content of this paper. The only contribution from industry was the donation of the custom programmed pacemakers that we received from St Jude Medical, now part of Abbott Laboratories.

## Data Availability

Data will be made available on request.
