# Supplementary material for: Resistance to atrial fibrillation domestication and mitochondrial dysfunction in sheep: a potential key role of the TCA cycle and mitochondrial redox state
Source: Redox Biol. 2026 Sep 10;97:104391. doi: 10.1016/j.redox.2026.104391 (PMC13602041; doi:10.1016/j.redox.2026.104391)
Supplement: Supplementary file 2 [file mmc2.docx]

**Resistance to Atrial Fibrillation Domestication and Mitochondrial Dysfunction in Sheep: a potential key role of the Krebs Cycle and mitochondrial redox state**

**Short title: AF resistance and mitochondrial adaptation**

**Author list:** Guido Caluori PhD ^a^, Stanley Nattel MD ^c^, Benoît Pinson PhD ^d,h^, Patrice Naud PhD ^c^, Bertrand Beauvoit PhD ^e^, Stéphane Claverol PhD ^f^, Fanny Vaillant PhD ^a^, Sabine Charron MsC ^a^, Florent Guilloteau BSc ^g^, Virginie Loyer ^a^, Marion Constantin MsC ^a^, Miruna Popa MD ^a^, Andrei Belykh PhD ^a^, Anaïs Rogowsky ^a^, Andreas Haeberlin MD, PhD ^a^, Sylvain Ploux MD, PhD ^a,b^, Hassan Adam Mahamat PhD ^a^, Rémi Dubois PhD ^a^, Bastien Guillot PhD ^a^, Philipp Krisai MD ^a^, Tsukasa Kamakura MD ^a^, Olivier Bernus PhD ^a^, Pierre Jaïs MD ^a,b^ , Pierre Dos Santos MD, PhD ^a,b^, Philippe Pasdois PhD ^a, h^

**Departments and Institutions:**

a. Univ. Bordeaux, INSERM, CRCTB, U 1045, IHU Liryc, F-33000 Bordeaux, France
b. CHU de Bordeaux, INSERM, U 1045, F-33000 Bordeaux, France
c. Department of Medicine and Research Center, Montreal Heart Institute and Université de Montréal, Montréal, Québec, Canada; Department of Pharmacology and Therapeutics, McGill University, Montréal, Québec, Canada; Institute of Pharmacology, West German Heart and Vascular Center, University of Duisburg-Essen, Essen, Germany.
d. Service d’Analyses Métaboliques- TBMCore, CNRS UAR 3427, Inserm US 005, Univ. Bordeaux, France
e. Univ. Bordeaux, INRAE, BFP, UMR 1332, F-33140 Villenave d'Ornon, France
f. Plateforme Protéome, Univ. Bordeaux, Bordeaux, France
g. Laboratoire de diagnostic moléculaire, hôpital Maisonneuve-Rosemont, Montréal, Québec, Canada
h. Univ. Bordeaux, CNRS, IBGC, UMR 5095, F-33000 Bordeaux, France

**Address for Correspondence:**

Philippe Pasdois. Université de Bordeaux, Institut de Biochimie et Génétique Cellulaires CNRS-UMR5095, 1, rue Camille Saint-Saëns, F-33077 Bordeaux, France. Tel : +33556999064. E-mail: [philippe.pasdois@u-bordeaux.fr](mailto:philippe.pasdois@u-bordeaux.fr)

**Article category**: Original article

***Summary of the main steps of the implantation protocol:***

1- **Preparation and monitoring of the sheep for the surgical procedure**

Sheep were prepared for the surgical procedure after an intramuscular injection of ketamine (20 mg/Kg), acepromazine (0.1 mg/Kg) and buprenorphine (9 µg/Kg). After installation of a venous line, at the level of the left front leg, anesthesia was induced by a bolus of propofol (1 mg/Kg). Sheep was then rapidly intubated and maintained under anesthesia using an equal mixture of air and oxygen supplemented by isoflurane (1.5 to 3%). Temperature, arterial pressure, PO_2_, PCO_2_, and ECG were monitored throughout the surgical procedure (Care Station 650, GE HealthCare).

2- **Preparation of the implantation area**

3- **Guide-wire 1 and 2 installations**

4- **First peelable introducer installed (7F for RV lead)**

5- **RV lead implantation and Screwing RV lead in the right ventricular free wall**

6- **Testing RV lead impedance and threshold (less than 1 mV) using the Merlin device from Abbott**
7- **Check RV lead position by X-Ray and suture the lead in place**

8- **Installation of the second peelable introducer (8F for RA lead)**

9- **IS1 lead and first telemetric leads (solid tip). The telemetric lead must be positioned at the entrance of the right atrial compartment**

10- **Screwing of the RA IS1 lead in the right atrial free wall + Testing RA lead for impedance and pacing threshold, must be under 1mV (pacing at 5mV for AF induction).**
11- **Preparation of the thorax area for implantation of the second telemetric lead subcutaneously. Use of X-Ray to define the position to cut for telemetric lead insertion. Forceps used as a spatial referential**

12- **Tunneling from the jugular site to the thorax. Necessary step to put the red telemetric lead in place (subcutaneously)**
13- **Insertion of the red lead from the jugular vein to the top of the thorax. IMPORTANT. Make a loop with the wire of the red electrode for optimal signal collection.**
14- **Test of Telemetric signal quality and then suture of the telemetric lead**

15- **Suturing of RA lead to the jugular vein**

16- **Creating a submuscular space in the neck to put the pacemaker and telemetric devices**
17- **Connection of the IS1 leads to the pacemaker.**

18- **Insertion of the devices in the submuscular lodge previously performed. Clean the IS1 leads telemetric leads, telemetric device & pacemaker with betadine before lodging them. Check signals with the Merlin device and induce some rapid atrial pacing to check that all devices are properly working.**
19- **For the telemetric device, the emitting antenna is localized on the written side of the implant. The antenna should be then positioned facing the skin and the muscle neck.**
20- **Before suturing inject some betadine into the wound**
21- **Suturing the muscle with non-absorbable yarn**
22- **Suturing of the skin with "resorbable" suture.**
23- **Telemetric signals check, P wave must have a minimum amplitude of 0.7mV at the end of the procedure to get a good SNR for the rest of the study.**
